# Bioinspired Lipoproteins of Furoxans–Gemcitabine Preferentially Targets Glioblastoma and Overcomes Radiotherapy Resistance

**DOI:** 10.1002/advs.202306190

**Published:** 2023-12-04

**Authors:** Maoyuan Sun, Honglei Xie, Wenli Zhang, Xianlu Li, Zhan Jiang, Yiyu Liang, Guanjian Zhao, Ning Huang, Jinning Mao, Guodong Liu, Zhiwen Zhang

**Affiliations:** ^1^ Department of Neurosurgery The Second Affiliated Hospital of Chongqing Medical University Chongqing 400016 China; ^2^ Institute of Pharmacology School of Pharmaceutical Sciences Shandong First Medical University & Shandong Academy of Medical Sciences 619 Changcheng Road Taian 271016 China; ^3^ Department of Radiology The Second Affiliated Hospital of Chongqing Medical University Chongqing 400016 China; ^4^ School of Pharmacy & Key Laboratory of Smart Drug Delivery (Ministry of Education) Fudan University Shanghai 201203 China; ^5^ Department of Oncology The Chongqing General Hospital Chongqing 400016 China; ^6^ Health Management Center The Second Affiliated Hospital Chongqing Medical University Chongqing 400016 China

**Keywords:** DNA repair, glioblastoma, lipoprotein, peroxynitrite, radiotherapy resistance

## Abstract

Radiotherapy (RT) resistance is an enormous challenge in glioblastoma multiforme (GBM) treatment, which is largely associated with DNA repair, poor distribution of reactive radicals in tumors, and limited delivery of radiosensitizers to the tumor sites. Inspired by the aberrant upregulation of RAD51 (a critical protein of DNA repair), scavenger receptor B type 1 (SR‐B1), and C‐C motif chemokine ligand 5 (CCL5) in GBM patients, a reduction‐sensitive nitric oxide (NO) donor conjugate of gemcitabine (RAD51 inhibitor) (NG) is synthesized as radio‐sensitizer and a CCL5 peptide‐modified bioinspired lipoprotein system of NG (C‐LNG) is rationally designed, aiming to preferentially target the tumor sites and overcome the RT resistance. C‐LNG can preferentially accumulate at the orthotopic GBM tumor sites with considerable intratumor permeation, responsively release the gemcitabine and NO, and then generate abundant peroxynitrite (ONOO^−^) upon X‐ray radiation, thereby producing a 99.64% inhibition of tumor growth and a 71.44% survival rate at 120 days in GL261‐induced orthotopic GBM tumor model. Therefore, the rationally designed bioinspired lipoprotein of NG provides an essential strategy to target GBM and overcome RT resistance.

## Graphical abstract

Inspired by the aberrant upregulation of DNA‐repairing RAD51, scavenger receptor B type 1 (SR‐B1), and C‐C motif chemokine ligand 5 (CCL5) in GBM patients, a reduction‐sensitive nitric oxide donor conjugate of furoxans–gemcitabine (NG) is rationally synthesized and a CCL5‐modified bioinspired lipoprotein system of C‐LNG is designed, which preferentially targets the orthotopic GBM tumors and profoundly circumvents the radiotherapy resistance.

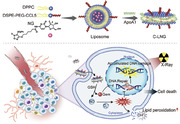

## 1 Introduction

Glioblastoma multiforme (GBM) is the most common and aggressive intracranial malignancy in adults.^[^ ^1^ ^]^ Radiotherapy (RT) is one of the current standard care for GBM patients, which can directly cause DNA double‐strand breaks (DSBs) or indirectly generate cytotoxic reactive oxygen species (ROS) to kill cancer cells.^[^ ^2^ ^]^ However, the therapeutic benefits of RT in GBM treatments remain dismal and greatly challenged by the RT resistance, which is related to the redundant DNA repair capacity, poor distribution of reactive radicals in tumors, and limited delivery of radio‐sensitizers to the tumor sites, etc.^[^ ^2^, ^3^ ^]^ Despite recent advances in cancer treatments, the median survival of GBM patients is only ≈12–14 months with a 2‐year overall survival of less than 20% and 5‐year survival of less than 5%.^[^ ^4^ ^]^ Therefore, novel strategies are highly desired to overcome the RT resistance for GBM treatment.

**Figure 1:**
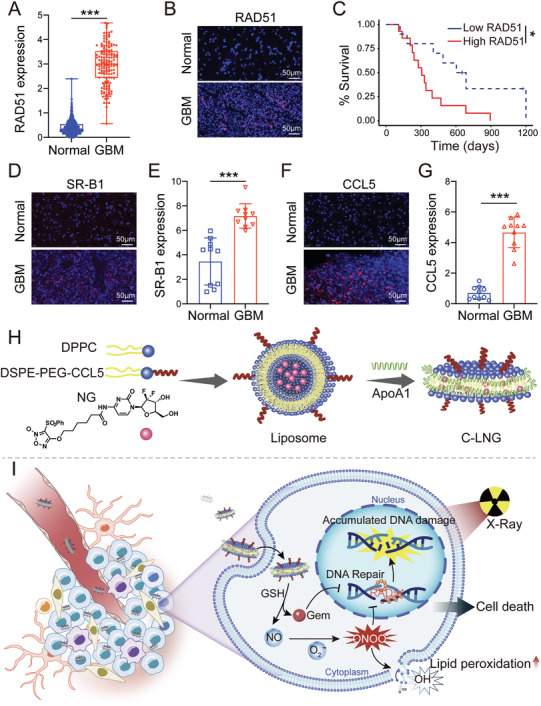
Analysis of RAD51, SR‐B1, and CCL5 in GBM patients and schematic illustration of designing C‐LNG to overcome RT resistance. A) A boxplot of RAD51 expression in GBM patients (*n* = 153) and healthy people (*n* = 1157) based on TCGA and GTEx databases. The data were transformed as log_2_(TPM + 1). B) Representative immunofluorescence images of RAD51 expression in tumor lesions and peritumoral tissues resected from GBM patients (*n* = 10). Scale bar: 50 µm. C) Survival curves of GBM patients with RT with high (*n* = 14) and low (*n* = 10) RAD51 expression. D) Representative immunofluorescence images and E) quantitative analysis of SR‐B1 expression tumor lesions and peritumoral tissues resected from GBM patients (*n* = 10). Scale bar: 50 µm. F) Representative immunofluorescence image and G) quantitative analysis for CCL5 expression in tumor lesions and peritumoral tissues resected from GBM patients (*n* = 10). Scale bar: 50 µm. H) Schematic illustration of the fabrication of C‐LNG. I) Preferential targeting C‐LNG to the orthotopic GBM tumors to potentiate RT efficacy by inhibiting DNA repair and inducing lipid peroxidation for GBM. Data are presented as mean ± SD. ^*^ *p* < 0.05, ^***^ *p* < 0.001.

The radiation treatments cause ubiquitous DNA damage in cancer cells, but the aberrantly upregulated DNA repair features attenuate DNA injuries and cause critical resistance to RT.^[^ ^5^ ^]^ RAD51 is a highly conserved protein that regulates DNA repair via homologous recombination, which promotes replication restart and provides error‐free repair of DSBs.^[^ ^6^ ^]^ Bioinformatics analyses of GBM patients revealed the abnormal upregulation of RAD51 in glioblastoma tissues when compared to that in normal brain tissues (**Figure** **1**A). Meanwhile, our data of GBM patients indicated that the RAD51 protein level was obviously elevated in glioblastoma tissues versus that in the peritumor tissues (Figure 1B; Figures S1 and S2, Supporting Information). The survival analysis indicated that the survival of GBM patients was negatively correlated with the RAD51 expression (Figure S3, Supporting Information). Extending this finding from GBM patients with RT, we certificated the increased expression of RAD51 in glioblastoma tissues after RT treatment (Figure S4, Supporting Information). More importantly, the survival analysis of GBM patients with RT showed that GBM patients with higher RAD51 expression had poorer survival (Figure 1C). As a result, the RAD51 inhibitor can be a promising candidate to suppress the DNA‐repairing activity for RT of GBM.

Although radiation treatments can generate abundant ROS radicals to damage the cancer cells, they are usually suffering from short lifetimes and limited diffusion distances, significantly restricting their spatio‐temporal distribution in tumors and compromising the cancer‐killing activity.^[^ ^7^ ^]^ Nitric oxide (NO) is a small membrane‐permeable signaling molecule, which exhibits a profound extension of biological half‐life (1–10 s) and diffusion distance (50–1000 µm) versus the common ROS radicals.^[^ ^8^ ^]^ NO is relatively stable and highly diffusible versus the superoxide anion (O_2_^−^) radicals, which can react with free O_2_^−^ radicals into potent cytotoxic peroxynitrite (ONOO^−^) radicals, demonstrating great potential to resolve the spatiotemporal distribution limitations of ROS radicals.^[^ ^9^ ^]^ Moreover, NO can be used to normalize the tumor vessel to relieve the hypoxic status in tumors and potentiate RT to produce more O_2_^−^ radicals, following more abundant ONOO^−^ radicals to induce robust DNA damage, making it an intriguing component of radiosensitizer.^[^ ^8^, ^10^ ^]^ On this basis, designing a NO‐donor conjugate of RAD51 inhibitor is plausible to resolve the challenges of DNA repair and poor intratumor distribution of radicals to potentiate RT treatment of GBM.

Of note, the radio‐sensitizing agents should be effectively delivered to the GBM tumor sites with considerable intratumor distribution profiles to exert therapeutic effects.^[^ ^11^ ^]^ However, GBM tumors are complex multifaceted microenvironments composed of multiple cellular and acellular components.^[^ ^12^ ^]^ Because of the intricate delivery barriers in GBM (e.g., blood–brain barrier, blood‐tumor barrier),^[^ ^13^ ^]^ the radio‐sensitizers are extremely difficult to accumulate at the GBM tumor sites and permeate the tumor tissues when they were administered as drug solution or drug‐loaded nanovesicle, raising an impressive need of designing delivery vehicles to target the GBM sites and improve the therapeutic outcome of RT for GBM treatment.

Within the GBM microenvironment, a variety of endogenous agents will be actively recruited to the tumor sites, which can effectively circumvent the delivery barriers and access the tumor tissues, providing an encouraging strategy to target GBM.^[^ ^14^ ^]^ On this rationale, we performed bioinformatics analyses of GBM patients and found that the expression of scavenger receptor class B type 1 (SR‐B1, a primary receptor of high‐density lipoprotein, HDL) and C‐C motif chemokine ligand 5 (CCL5) in GBM tissues are significantly higher than that in normal brain tissues (Figures S5 and S6, Supporting Information). As proof of principle demonstration, we investigated the expression of SR‐B1 and CCL5 in GBM samples and found that the protein levels of SR‐B1 and CCL5 were obviously elevated in tumor tissues versus in the peritumor tissues (Figure 1D–G; Figure S1, Supporting Information). HDL are endogenous nanosized vehicles composed of versatile phospholipids, cholesterol ester, and apolipoprotein A‐1 (ApoA1), which can load versatile therapeutic agents and represent an effective tumor‐targeted delivery vehicle.^[^ ^8^, ^15^ ^]^ Likewise, CCL5, abundantly secreted in the GBM sites, can be released into the peripheral circulation system and mediate the chemotaxis of versatile accessory cells (e.g., monocytes, lymphocytes) to the tumor sites.^[^ ^16^ ^]^ Accordingly, we suppose that the combination of HDL and CCL5 can facilitate the targeted delivery of therapeutic agents to the GBM sites to achieve anti‐tumor effects.

For these perspectives, we herein synthesized a reduction‐sensitive NO donor conjugate of furoxans–gemcitabine (Gem, a RAD51 inhibitor) (NG) as radio‐sensitizer and then rationally designed a CCL5 peptide‐modified bioinspired lipoprotein system of NG (C‐LNG), aiming to preferentially target the GBM tumor sites and potentiate the RT efficacy (Figure 1H). In this design, the combination of CCL5 peptide and bioinspired lipoprotein system was used to target the GBM sites, while NG was used as a radio‐sensitizer for overcoming RT resistance. Upon their delivery to the GBM tumor tissues, Gem and NO would be responsively released in response to glutathione (GSH), which could respectively inhibit DNA repair and generate abundant cytotoxic ONOO^−^ upon X‐ray radiation, thereby improving RT treatment of GBM (Figure 1I). In this manuscript, we characterized the drug‐releasing capacity of C‐LNG, investigated their targeting behavior to the orthotopic GBM tumor, evaluated the spatiotemporal distribution of ONOO^−^ radicals in tumor tissues, and explored their therapeutic benefits in two orthotopic GBM tumor models to validate the effectiveness of this design on overcoming RT resistance.

## 2 Results and Discussion

### 2.1 Characterizations of C‐LNG

Based on the rationale of overcoming RT resistance by inhibiting DNA repair and enhancing spatiotemporal distribution of cytotoxic radicals, we first synthesized the NG conjugate by linking the NO‐donor group of furoxans with the RAD51 inhibiting group of Gem. In contrast, a counterpart lipophilic conjugate of Gem without NO donor was synthesized by linking Gem with tetradecyl succinic anhydride (named C_14_‐Gem), wherein Gem is linked with other segments at the ‐NH_2_ terminal to ensure their comparable link site to the NG conjugate. Meanwhile, we developed a 1,2‐distearoyl‐sn‐glycero‐3‐phosphoethanolamine–N‐poly(ethylene glycol)2000 (DSPE–PEG)–CCL5 (DSPE–PEG–CCL5) derivative by conjugating the thiol group of CCL5‐mimetic peptide (sequence, CFPYIARPLPRAHIKEYFY) to DSPE–PEG–maleimide. The characterizations of ApoA1 and CCL5 peptides are provided in Figures S7 and S8 (Supporting Information). Detailed information on synthesis procedures and characterizations of NG, C_14_‐Gem, and DSPE‐PEG‐CCL5 were provided in the supporting information (Figures S9–S11, Supporting Information).

**Figure 2:**
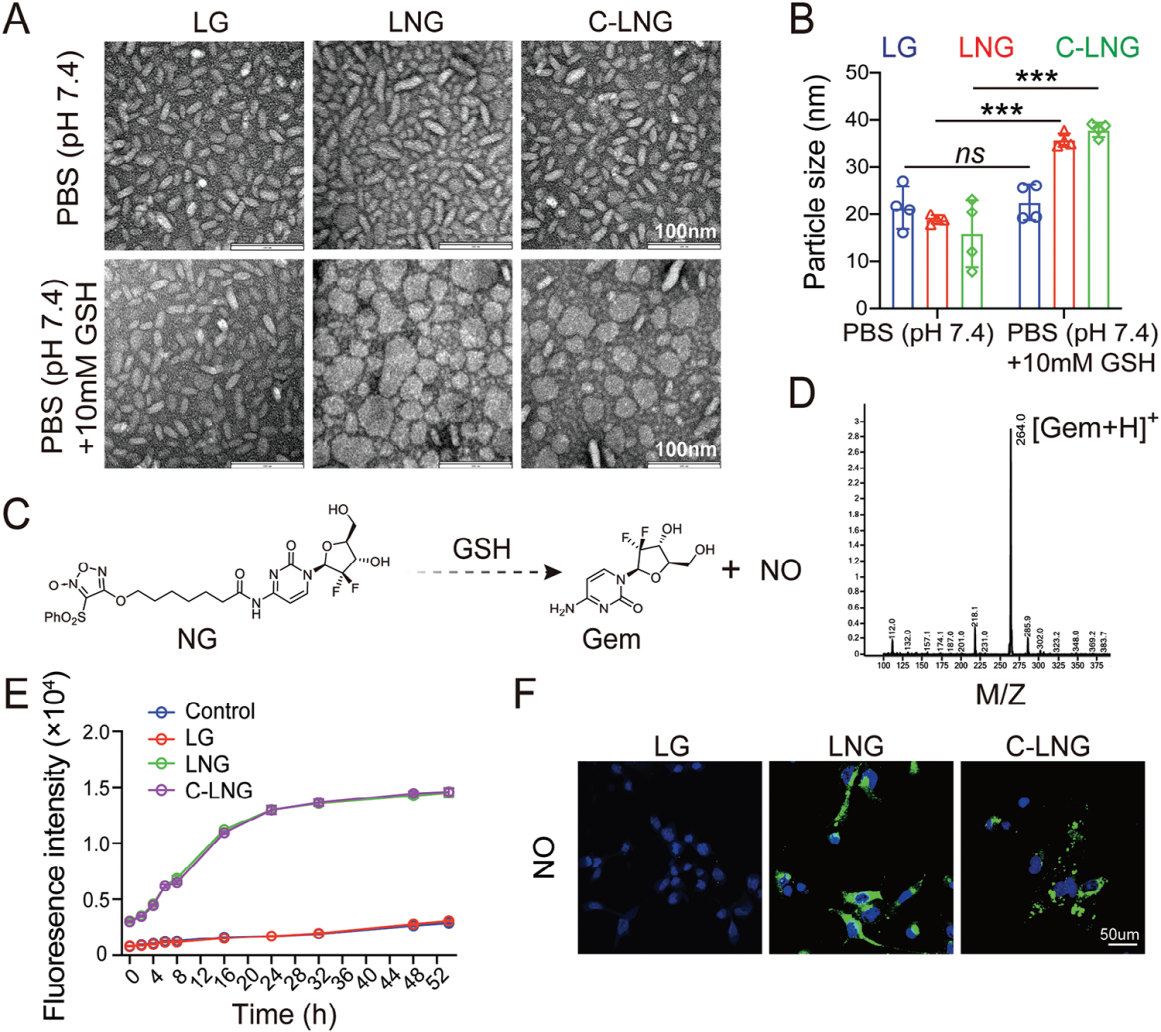
Characterizations of C‐LNG. A) Representative TEM images of LG, LNG, and C‐LNG before and after incubation in PBS (pH 7.4) + 10 mm GSH) for 48 h. scale bar = 100 nm. B) Mean particle size of LG, LNG, and C‐LNG in media with or without 10 mM GSH (*n* = 4). C) GSH‐responsive degradation of NG into gemcitabine and NO. D) Typical MS profile of released gemcitabine. E) Release profile of NO from LG, LNG, and C‐LNG upon their incubation in PBS (pH 7.4) + 10 mm GSH (*n* = 4). F) NO production in GL261 cells from the LG, LNG, or C‐LNG treated groups, wherein NO was denoted as green fluorescence signals. scale bar, 50 µm. Data are presented as mean ± SD. ^***^ *p* < 0.001.

The bioinspired lipoprotein system of C‐LNG was fabricated with 1, 2‐Dipalmitoyl‐sn‐glycero‐3‐phosphocholine (DPPC), DSPE‐PEG‐CCL5, NG, and ApoA1 mimic peptide (PVLDLFRELLNELLEALKQKLK). In contrast, a counterpart lipoprotein formulation of NG without CCL5 peptide (termed as LNG) was prepared with DPPC, DSPE‐PEG, NG, and ApoA1 peptide, and a control lipoprotein formulation of C_14_‐Gem without CCL5 peptide (termed as LG) was composed of DPPC, DSPE‐PEG, C_14_‐Gem, and ApoA1 peptide. The loading of these active agents in the bioinspired lipoprotein system was determined by the high‐performance liquid chromatography (HPLC) method. The encapsulation efficiency of LG, LNG, and C‐LNG was respectively 89.77 ± 3.06%, 92.11 ± 1.93%, 93.16 ± 0.73%, with the drug loading (DL) capacity of 8.98 ± 0.31%, 8.37 ± 0.18%, 8.47 ± 0.07%, indicating the efficient loading of these active agents in the bioinspired lipoprotein system. The morphologies of C‐LNG, as well as the counterpart LG and LNG, were visualized using transmission electron microscopy (TEM) (**Figure** **2**A). The TEM images showed that all of the LG, LNG, and C‐LNG were homogenous nanometer‐sized discoidal particles, suggesting their similarity to the HDL system. The DLS results further indicated mean diameter of LG, LNG, and C‐LNG is 21.48 ± 4.49, 18.96 ± 0.87, and 15.93 ± 7.11 nm, respectively (Figure 2B). When they were incubated in phosphate‐buffered saline (PBS, pH 7.4) and PBS (pH 7.4) + 10% fetal bovine serum (FBS) for 48 h, the mean diameter was not obviously changed (Figures S12 and S13, Supporting Information). Moreover, in the C‐LNG system, the EE value of NG was over 90% upon their incubation in the PBS (pH 7.4) and PBS (pH 7.4)+10% FBS at 37 °C for 48 h (Figure S14, Supporting Information). These data confirmed their good stability in the mimicked physiological fluids and their feasibility for in vivo delivery.

Then, we measured the reduction‐sensitive properties of these three lipoprotein formulations to the reductive GSH by incubating them in PBS containing 10 mm GSH. After 48 h of incubation, the TEM images indicated that the NG‐loaded LNG and C‐LNG were changed from discoidal particles into irregular or spherical shapes with a significant difference, and the DLS analysis confirmed the size enlargement with a mean diameter of 35.76 ± 1.46 nm for LNG and 37.95 ± 1.48 nm for C‐LNG, respectively (Figure 2A,b; Figure S12, Supporting Information). However, the morphologies and mean particle size of LG were rarely changed (Figure 2A,B; Figure S13, Supporting Information). To characterize the time‐dependent responsive drug release profiles from C‐LNG, the EE value of NG was monitored after incubation in PBS (pH 7.4) +10 mm GSH at 37°C for 48 h. The EE value of NG from C‐LNG in PBS (pH 7.4) +10 mm GSH was gradually reduced with time at 4 h of incubation, and less than 10% in PBS (pH 7.4) +10 mm GSH at 48 h of incubation, suggesting the reduction‐responsive release profiles from the C‐LNG. By comparing their formulations to LG, NG could be a major contributor to the GSH‐responsiveness of LNG and C‐LNG.

Then, we measured the possible degradation of NG from C‐LNG after their incubation with PBS containing 10 mm GSH (Figure 2C–E). The typical peak of [Gem+H]^+^ was readily detected by the electrospray ionization mass spectrometry (ESI‐MS) analysis, which effectively verified the degradation of NG into active Gem (Figure 2D; Figure S15, Supporting Information). Meanwhile, the NO release from these three formulations was detected using a typical NO fluorescence probe of 4,5‐diamino‐rhodamine B (DAR‐1) (Figure 2E). The fluorescence signals of NO could be detected from LNG and C‐LNG at 4 h of incubation in PBS (pH 7.4) + 10 mm GSH and then gradually increased until 24 h, but were barely detected in the control and LG groups (Figure 2E), confirming the NO‐producing activity of NG in the LNG and C‐LNG groups. The NO‐generating activity of LNG and C‐LNG was further evaluated in GL261 cells under a confocal laser scanning microscope (CLSM). The green fluorescence signals could be readily visualized in cancer cells treated with LNG and C‐LNG but were hardly observed in the LG‐treated group (Figure 2F). The flow cytometer analysis also confirmed the higher NO‐producing capacity in the LNG and C‐LNG treated GL261 cells with no difference between them (Figure S16, Supporting Information). In addition, the intracellular GSH levels were respectively reduced by 71.36% and 69.42% in LNG and C‐LNG‐treated GL261 cancer cells, but rarely changed in the LG‐treated cells, suggesting the important role of GSH in activating NO release from LNG and C‐LNG in cancer cells (Figure S17, Supporting Information). These data verified the GSH‐responsive drug release profiles of C‐LNG, demonstrating great potential to release Gem and NO in cancer cells to exert therapeutic effects.

### 2.2 In Vitro Therapeutic Effects of C‐LNG in GL261 Cancer Cells

**Figure 3:**
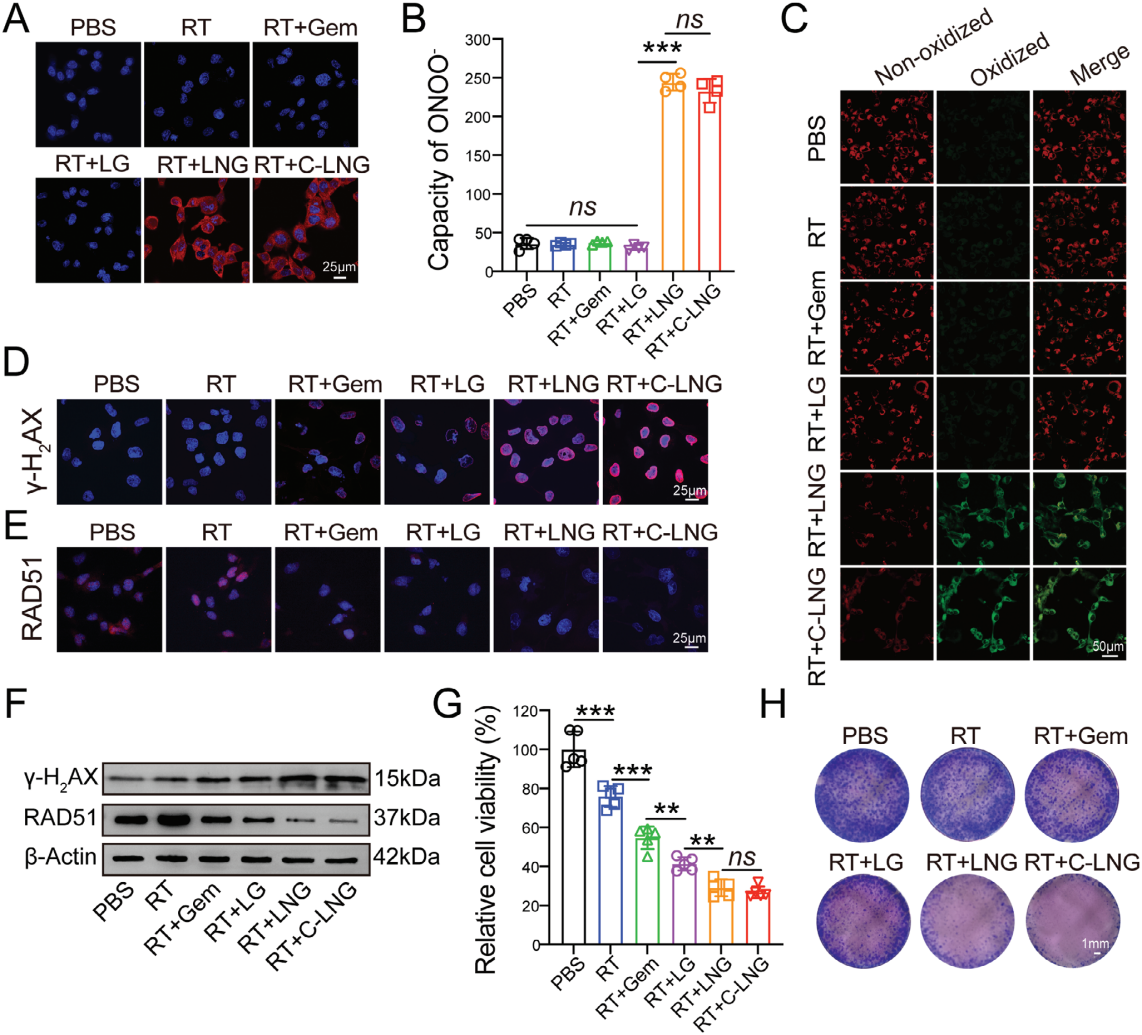
In vitro therapeutic efficacy in GL261 cells. A) Representative CLSM images of GL261 cells with various formulation and their combination with RT, wherein ONOO^−^ radicals were denoted as red signals. Scale bar: 25 µm. B) Quantified production of ONOO^−^ from each treatment (*n* = 4). C) CLSM images of lipid peroxidation in GL261 cells from each treatment using a lipid peroxidation probe of ^11^C‐BODIPY, wherein the oxidized lipids were denoted as green fluorescence signals. Scale bar: 50 µm. D) γ‐H_2_AX expression in GL261 cells from diverse treatments. Scale bar: 25 µm. E) RAD51 expression in GL261 cells from diverse treatments. Scale bar: 25 µm. F) Western blot analysis of γ‐H_2_AX and RAD51 expression in cells from each treatment. G) Relative cell viabilities of GL261 cells from each treatment (*n* = 5). H) Colony formation assays of GL261 cells from each treatment. Scale bar: 1 mm. Data are presented as mean ± SD. ^**^ *p* < 0.01, ^***^ *p* < 0.001.

The ONOO^−^ production in GL261 cancer cells was visualized by CLSM detections and flow cytometer examinations. In the CLSM images, the red fluorescence signals of ONOO^−^ were obviously detected in cells treated with LNG and C‐LNG plus X‐ray radiation (2 Gy) (RT+LNG, RT+C‐LNG), but rarely observed in other groups (**Figure** **3**A). The quantified results indicated that the fluorescence intensity of ONOO^−^ in the RT+LNG and RT+CLNG treated cells was profoundly improved by 7.92‐ and 7.55‐fold versus that in cells treated with the counterpart LG plus RT (RT+LG), but no significant difference occurred between them (Figure 3B). These data confirmed the efficient production of ONOO^−^ radicals in the RT+LNG and RT+CLNG treated cells, which could be attributed to the loaded NG in the bioinspired lipoprotein system.

In view of the abundant production of ONOO^−^ radicals, we measured their impacts on the promoting lipid peroxidation of cell membranes using a C_11_‐BODIPY probe. In the captured CLSM images, the oxidized lipids in the cell membrane were denoted as green fluorescence signals while the non‐oxidized lipids were listed as red fluorescence signals. As shown in Figure 3C, the oxidized form of lipids was readily visualized in the RT+LNG and RT+C‐LNG treated cells with strong green fluorescence signals but barely detected in other groups. Meanwhile, the incidence of lipid peroxidation in cells treated with RT+LNG and RT+C‐LNG was confirmed by flow cytometer examinations (Figure S18, Supporting Information). Then, we measured their effects on inducing DNA damage in GL261 cancer cells by detecting the expression of γH_2_AX, a typical marker of DNA injury. The γH_2_AX signals in the nuclei were evidently visualized in the RT+LNG and RT+C‐LNG treated cells with the fluorescence intensity much stronger than those in other groups (Figure 3D). The western‐blot analysis indicated that the protein signals of γH_2_AX in the RT+LNG and RT+C‐LNG treated group were much higher than those in other groups (Figure 3F). In contrast, the expression of the DNA‐repair protein of RAD51 was measured by immunofluorescence assays and western‐blot analysis. The red fluorescence signals of RAD51 in the nuclei were readily observed in the control group and cell with RT alone but obviously reduced in the other groups (Figure 3E). Moreover, when comparing to the negative control, the protein signals of RAD51 were unexpectedly increased in the cells treated with RT alone, but significantly reduced after their combination with Gem and LG, and further weakened in cells treated with RT+LNG and RT+C‐LNG (Figure 3F). These data effectively verified the merits of RT+C‐LNG treatment in promoting lipid peroxidation, potentiating DNA damage, and inhibiting DNA repair activity, thereby demonstrating great potential to kill cancer cells.

Consequently, we evaluated the efficacy of RT+C‐LNG treatment in suppressing the viability of GL261 cancer cells. When comparing to the negative control, the cell viability was significantly inhibited by RT and RT+Gem treatments and respectively reduced to 29.08 ± 4.34% by the RT+LNG treatment and 26.82 ± 2.97% by the RT+C‐LNG treatment (Figure 3G). In addition, the C‐LNG treatment caused significant toxicity in GL261 cancer cells and no obvious toxicity in bEnd.3 cells at concentration < 40 µg mL^−1^ of NG (Figure S19, Supporting Information). The GL261 cancer cells were proliferating cells with more frequent DNA replication than normal endothelial bEnd.3 cells, which could be more prone to be killed by the DNA‐damaging Gem via inhibiting DNA chain elongation during DNA replication, thereby resulting in the higher cytotoxicity of C‐LNG in GL261 cancer cells. The colony formation assays further confirmed the efficient anti‐proliferation effects of RT+C‐LNG treatment. The clustered signals of cell colony were scarcely observed in the RT+LNG and RT+C‐LNG treated groups, but considerably detectable in other groups (Figure 3H). These results verified the effects of RT+C‐LNG treatment on inhibiting cell viability and proliferation. By comparing the formulation of C‐LNG with counterpart LNG and LG, NG could be an effective radiosensitizer to synergize RT for GBM treatment.

### 2.3 Tumor Targeting and Intratumor Distribution in Orthotopic GBM Tumor Model

**Figure 4:**
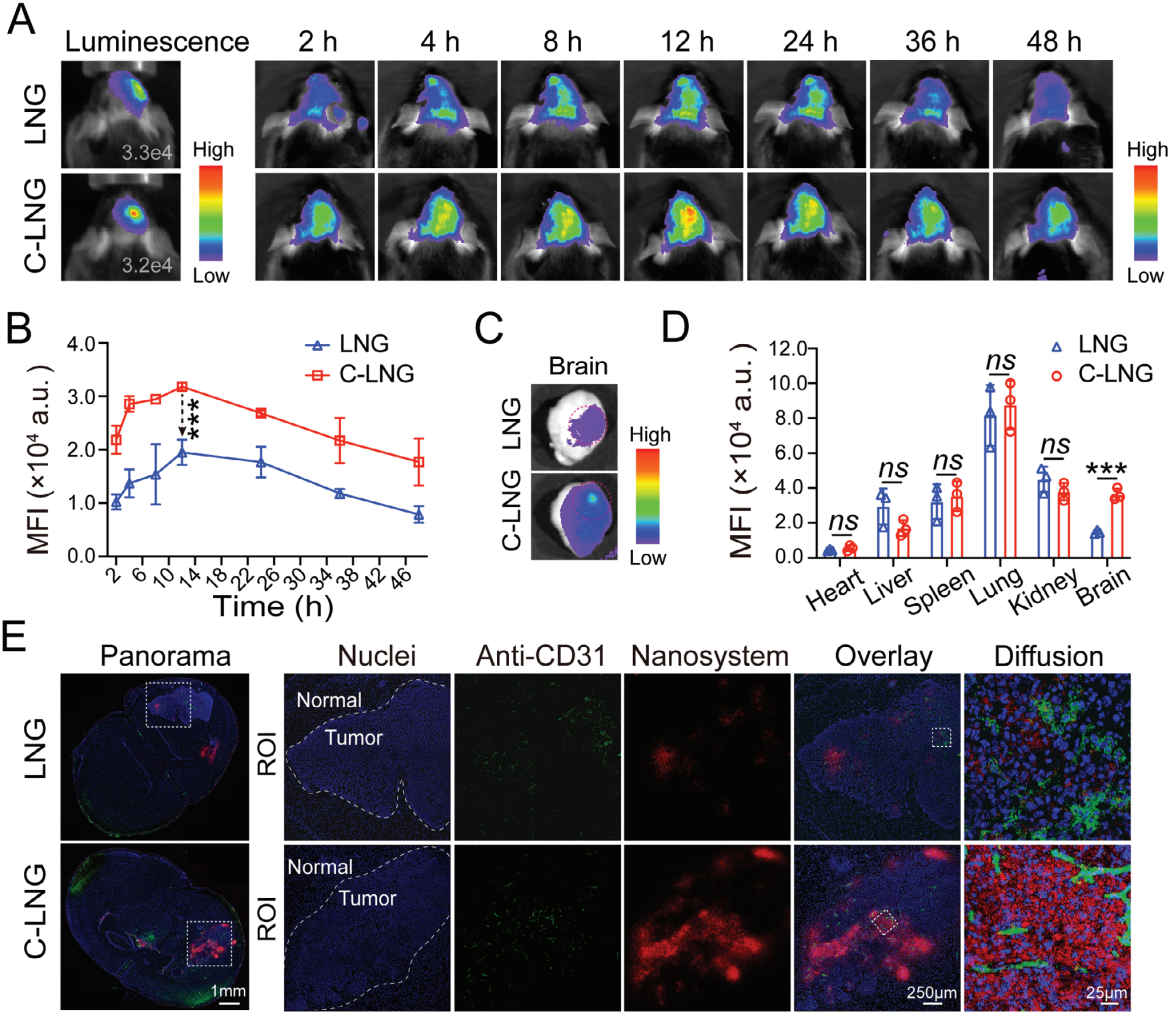
In vivo targeting of C‐LNG to orthotopic GBM tumor lesions. A) In vivo distributions of DiD‐loaded LNG and C‐LNG to the brain tissues with GL261‐luc induced orthotopic GBM tumor model. The fluorescence signals were detected at certain time intervals post‐injection. B) Quantified fluorescence signals in the brain tissues (*n* = 3). C) Representative ex vivo fluorescence images of the brain tissues. D) Quantitative analysis of DiD‐loaded LNG and LNG in the major organs at 12 h after i.v. injection (*n* = 3). E) Distribution of DiD‐loaded LNG and C‐LNG in the whole brain tissues, access to the GBM tumor lesions, and diffusion from blood vessels into tumor parenchyma. Data are presented as mean ± SD. ^***^ *p* < 0.001.

To achieve the radio‐sensitizing effects, the preferential targeting of C‐LNG to the orthotopic GBM tumor sites and their penetration into deep tumor regions could be an essential prerequisite.^[^ ^13^, ^17^ ^]^ Leveraging on the upregulated SR‐B1 and CCL5 in tumor tissues, the combination of ApoA1‐based artificial lipoprotein and the N‐terminal peptide of CCL5 in C‐LNG was supposed to promote their targeted delivery to the orthotopic GBM tumor tissues in an endogenous targeting manner. To verify this hypothesis, the targeting and distribution behavior of C‐LNG and counterpart LNG was determined in an orthotopic GBM tumor model, which was developed by orthotopic injection of GL261 cancer cells with stable expression of luciferase (GL261‐luc). The formation of orthotopic GBM tumors was monitored by bioluminescence assays prior to the experiments (**Figure** **4**A). Both C‐LNG and counterpart LNG were labeled with a hydrophobic fluorescence probe of DiIC18(5) (DiD) for the detections. The in vivo imaging indicated the fluorescence signals of C‐LNG and counterpart LNG could be detectable in the brain tissues at 2 h post‐injection, and maximized at 12 h (Figure 4A,B), indicating their effective accumulation at the brain tissues with orthotopic GBM tumors. At certain time points, the fluorescence intensity in the brain from C‐LNG was notably higher than that from counterpart LNG formulation (Figure 4A,B), suggesting the better targeting capacity of C‐LNG over LNG to the brain tissues with orthoptic GBM tumor. The preferential accumulation of C‐LNG in the brain tissues with orthotopic GBM tumor was further confirmed by ex vivo imaging of the major organs at 12 h of injection (Figure 4C; Figure S21, Supporting Information). The quantified results indicated that the fluorescence signals in the brain tissues from the C‐LNG‐treated group were improved 2.45‐fold versus that from the LNG‐treated group (Figure 4D). In addition, we investigated the distribution of C‐LNG in the brain tissues of healthy mice or GL261‐induced orthoptic GBM tumor models. The fluorescence signals of C‐LNG were obviously detected in the brain tissue of the orthotopic GBM tumor model but merely visualized in that of healthy mice, suggesting the tumor‐triggered endogenous targeting capacity of the C‐LNG (Figure S22, Supporting Information). In addition, both LNG and C‐LNG exhibited comparable circulation profiles in the blood samples from healthy mice, with a half‐life time of 11.72 h for C‐LNG and 9.03 h for LNG (Figure S20, Supporting Information). As a result, these data confirmed the preferential targeting of C‐LNG to the brain tissues with orthotopic GBM tumors.

Then, we evaluated their targeted delivery to the tumor lesions in the brain tissues by the CLSM imaging method. In the transverse profiles of the whole brain with tumor lesions, the red fluorescence signals of C‐LNG could be considerably and extensively visualized in the GBM tumor lesions, but rarely detectable in the adjacent normal brain tissues (Figure 4E). In contrast, those of counterpart LNG could be detected in the GBM tumor lesions and normal regions distant from the tumor lesions. In the enlarged images of the GBM tumor lesions, C‐LNG could be efficiently distributed in the whole GBM tumor lesion while LNG was mainly located in the exterior region of the tumor lesions (Figure 4E). Moreover, the signals of C‐LNG could be observed in areas outside the tumor vessels with a fluorescence intensity much stronger than those of LNG, suggesting the efficient diffusion of C‐LNG from tumor vessels into tumor parenchyma (Figure 4E). Moreover, the quantified results indicated that the Gem amount in the GBM tumors from the C‐LNG treated group was enhanced 2.51‐fold versus that from the LNG group (Figure S20, Supporting Information). These results effectively evidenced the preferential and efficient targeting capacity of C‐LNG to the orthotopic GBM tumors, which would facilitate their access to various cellular compartments in tumors to achieve therapeutic effects. By analyzing the formulation of C‐LNG and counterpart LNG, the CCL5‐mimic peptide could be the primary contributor to the efficient tumor targeting profile. The incorporation of the CCL5 peptide in the C‐LNG could integrate the chemotactic activity of CCL5 and the features of the HDL system into a bioinspired drug delivery vehicle, thereby providing an encouraging strategy to target the orthotopic GBM tumors in the brain tissues.

**Figure 5:**
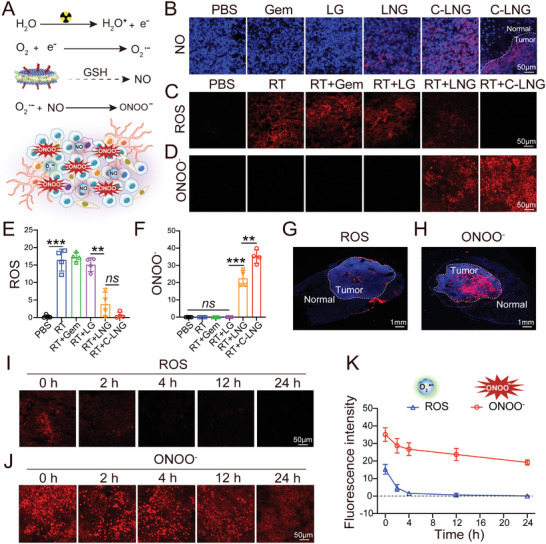
Spatio‐temporal distribution of reactive radicals in orthotopic GBM tumor. A) Schematic illustration of ONOO^−^ production upon X‐ray radiation (2 Gy). B) NO productions in tumors from each group. Scale bar: 50 µm. C) Generation of ROS and D) ONOO^−^ in tumors from each treatment. Scale bar: 50 µm. E) Quantified results of ROS in tumors from each treatment (*n* = 4). F) Quantified results of ONOO^−^ in tumors from each treatment (*n* = 4). G) Spatial distribution in the whole brain of ROS from the RT+LG group. H) Spatial distribution in the whole brain of ONOO^−^ from the RT+C‐LNG group. The white dashed lines represent tumor boundaries differentiated by cell density. I) Temporal distribution of ROS in tumors at certain time points. Scale bar: 50 µm. J) Temporal distribution of ONOO^−^ in tumors at certain time points. Scale bar: 50 µm. K) The quantified analysis of ROS and ONOO^−^ in tumors at certain time points (*n* = 4). Data are presented as mean ± SD. ^**^ *p* < 0.01, ^***^ *p* < 0.001.

Upon radiation treatment, the excited electrons could react with O_2_ to generate superoxide anion (O_2_^•−^), and subsequently react with the generated NO molecules into highly reactive cytotoxic ONOO^−^ radicals,^[^ ^8^, ^9^, ^18^ ^]^ which would exhibit longer lifetime and diffusion distance in tumors to exert the cancer‐killing effects (**Figure** **5**A). The production of NO and their distribution in orthotopic GBM tumors were measured by CLSM imaging (Figure 5B). The red fluorescence signals of NO were obviously observed in the LNG and C‐LNG treated groups, but undetected in other groups. Of note, in the C‐LNG treated orthotopic GBM tumor model, the NO signals were preferentially located in the GBM tumor lesions but undetectable in the peritumor regions. These data confirmed the efficient NO production and their precise distribution in the orthotopic GBM tumor lesions.

When they were exposed to X‐ray radiation at 2 Gy, the production of cytotoxic ROS and ONOO^−^ radicals from each treatment was determined. As shown in Figure 5C–F, the signals of ROS in orthotopic GBM tumor lesions were mainly detected in the RT alone, RT+Gem, and RT+LG treated groups, but obviously decreased in the LNG and C‐LNG treated groups (Figure 5C). In contrast, the signals of ONOO^−^ radicals in orthotopic GBM tumor lesions were readily visualized in the LNG and C‐LNG treated groups, but rarely detectable in other groups (Figure 5D). The quantified results indicated the signals of ROS in the C‐LNG treated group were only 4.10%, 4.46%, and 17.69% of those in the RT alone, RT+LG, and RT+LNG treated groups, respectively (Figure 5E). In contrast, the signals of ONOO^−^ radicals in the C‐LNG treated tumors were profoundly higher than those in other groups and significantly increased 1.56‐fold versus those in the RT+LNG treated group (Figure 5F).

Regarding the production of ROS in the RT+LG group and the ONOO^−^ radicals in the RT+C‐LNG group, we examined the spatiotemporal distribution of these radicals in the orthotopic GBM tumor lesions (Figure 5G–K). In the transverse profiles of the whole brain tissues, the signals of ONOO^−^ radicals in the RT+C‐LNG group were largely detected in the GBM tumor lesions with strong fluorescence intensity but invisible in the normal brain tissues, however, those of ROS in the RT+LG group were sparsely distributed in the tumor lesions and were observable in the normal brain tissues (Figure 5G,H), demonstrating the precise spatial distribution of the ONOO^−^ radicals in orthotopic GBM tumors treated with RT+C‐LNG.

Afterward, we measured the temporal distribution profiles of the ONOO^−^ radicals in the RT+C‐LNG group and the RT+LG group at different time points after radiation. As shown in Figure 5I,J, the red fluorescence signals of ONOO^−^ radicals in the RT+C‐LNG treated orthotopic GBM tumors were readily visualized and slightly reduced with time, which could be retained at a high level even at 24 h of radiation. In contrast, the red signals of ROS in the RT+LG treated GBM tumors could be visualized at 2 h post‐radiation, but rapidly decreased with time, and became invisible at 4 h of radiation. Moreover, the image analysis indicated that the mean fluorescence intensity of ONOO^−^ radicals in the RT+C‐LNG treated tumors was obviously higher than that of ROS in the RT+LG treated tumors at each time point of radiation (Figure 5K). As a result, the design of the ONOO^−^ radicals in the RT+C‐LNG treated group exhibited superior efficacy in improving spatiotemporal distribution profiles over the common ROS in the RT+LG treated tumors, providing an essential opportunity to kill cancer cells. The higher level of ONOO^−^ signals at the tumor sites from the C‐LNG treated group could be attributed to the notable tumor targeting capacity, sustainable production from the reaction of released NO with O_2_^−^ radicals, and the higher steady‐state concentration.^[^ ^9d,e^ ^]^

### 2.4 In Vivo Therapeutic Benefits of C‐LNG in Orthotopic GBM Tumor Models

**Figure 6:**
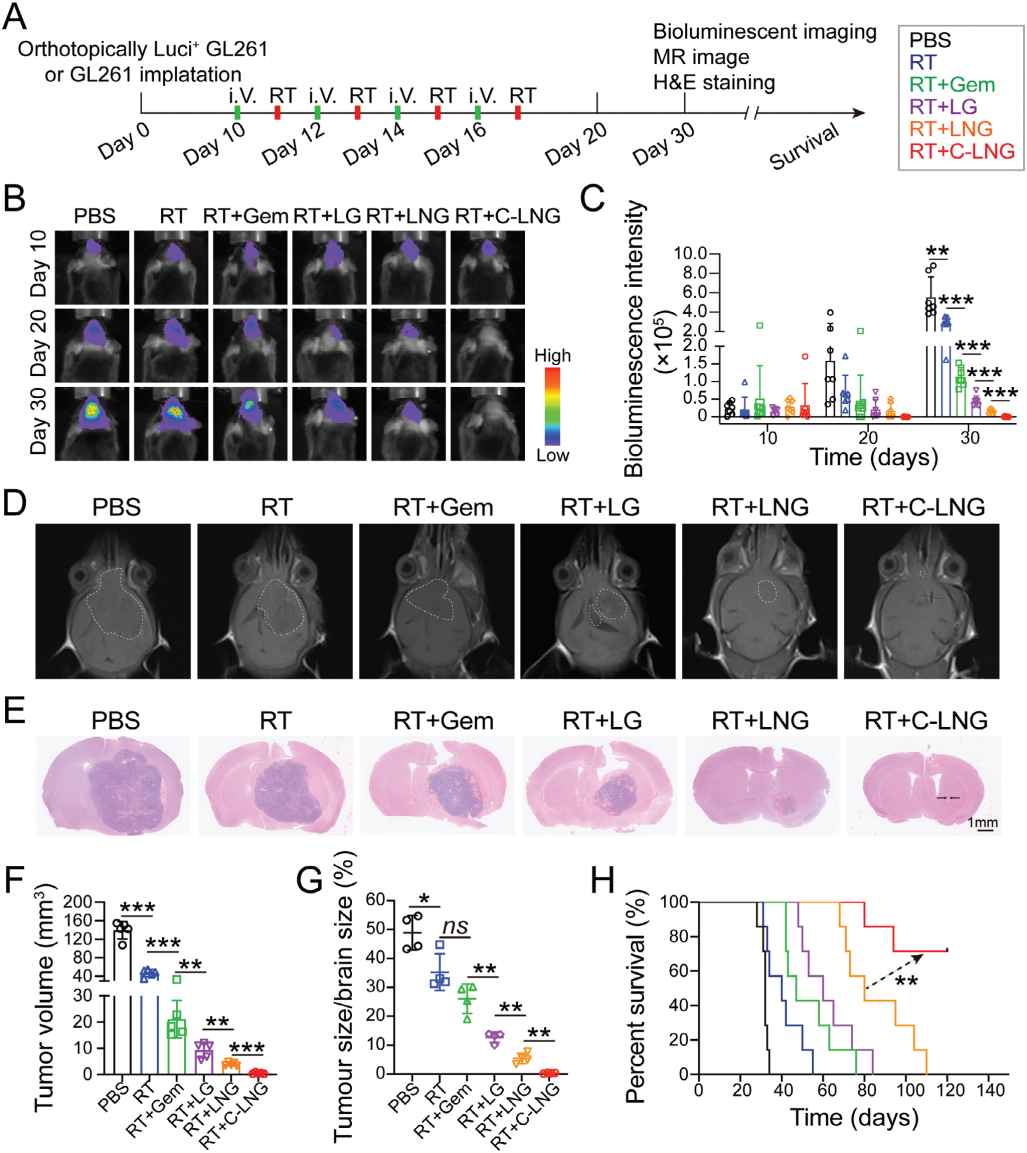
In vivo therapeutic efficacy of C‐LNG on improving RT against orthotopic GBM. A) Schematic illustration of the drug treatment schedule in GL261‐luc induced orthotopic GBM tumor models. The tumor‐bearing mice were respectively injected with free Gem, LG, LNG, and C‐LNG at 5 mg kg^−1^ of NG or a comparable dose on days 10, 12, 14, and 16 and subsequently exposed to X‐ray radiation after 12 h post‐injection. B) Bioluminescence images of the brain tissues from each treatment at indicated time points. C) Quantified results of the bioluminescence signal intensity in the brain tissues from each treatment (*n* = 7). D) T1‐weighted MR images of the mouse head on day 30 post various treatments. E) Histological examinations of the whole brain tissues from each treatment, wherein the tumor lesions were denoted as cell clusters with darkly stained nuclei. F) Tumor volumes from each treatment by MRI imaging (*n* = 5). G) Quantitative analysis of the tumor size/brain size by image J according to the H&E staining images (*n* = 4). H) Kaplan–Meier survival curves of mouse models from each treatment (*n* = 7). Data are presented as mean ± SD. ^*^ *p* < 0.05, ^**^ *p* < 0.01, or ^***^ *p* < 0.001.

Encouraged by the prevailing effects of C‐LNG in preferentially targeting the GBM tumor and improving intratumor distribution profiles of the highly reactive ONOO^−^ radicals, we measured their efficacy on depressing tumor growth and extending survivals in GL261‐luc induced orthotopic GBM tumor models after multiple treatments (**Figure** **6**A). The radiation treatments were performed at a dose of 2 Gy at 12 h post‐injection, and the extent of tumor progression was monitored by bioluminescence imaging assays at certain time points of treatments (Figure 6B; Figure S24, Supporting Information). The signals of GL261‐luc cancer cells in the brain tissues were continuously increased with time in the control and RT‐alone groups, but significantly delayed in the RT+Gem and RT+LG treated groups. In the RT+LNG treated group, the signals of GL261‐luc cancer cells in the brain tissues were maintained at extremely low levels and hardly increased with time, indicating the effective retardation of tumor growth by the RT+LNG treatment. Strikingly, when mice were treated with RT+C‐LNG, the signals of GL261‐luc cancer cells in the brain tissues were hardly detected at day 20 and day 30 of treatment, indicating the superior efficacy of C‐LNG on potentiate RT to inhibit tumor growth (Figure 6B,C). Meanwhile, we quantified the tumor growth index (TGI) by comparing the mean bioluminescence intensity in the brain tissues at day 30 to that at day 10 (the initial time point of treatment) to evaluate the tumor‐inhibitory effects. The quantified analysis indicated that the TGI value was 22.93 for the control and 13.33 for RT alone, indicating the aggressive tumor growth and the poor validity of the RT‐alone treatment. In contrast, the TGI was reduced to 2.18 for RT+Gem, 2.83 for RT+LG, and 0.53 for RT+LNG, indicating the significant delay of tumor growth by these treatments. Importantly, the TGI was only 0.015 when RT was combined with C‐LNG, which was profoundly lower than that in other groups. These results certificated the prominent efficacy of the RT+C‐LNG treatment in suppressing tumor growth. In addition, the biosafety assays indicated that the numbers of red blood cells (RBC), white blood cells (WBC), and platelets (PLT) as well as the levels of alanine transaminase (ALT), aspartate aminotransferase (AST) and creatinine (CREA) in the blood samples from each group were all in the normal ranges, and the histological examinations showed no histological changes were detected in the major organs (Figure S25, Supporting Information), confirming the good biocompatibility of these formulations.

Moreover, we utilized magnetic resonance imaging (MRI, T1‐weighted image) to monitor the tumor volume at day 30 of treatment (Figure 6D; Figure S26, Supporting Information). The GL261‐luc‐induced orthotopic GBM tumors were almost overgrown in the frontal‐parietal lobe at 30 days in the control group with a tumor volume of 139.59 ± 18.73 mm^3^. The tumor volume was respectively reduced to 47.07 ± 7.78 mm^3^ for RT alone, 21.14 ± 7.15 mm^3^ for RT+Gem, 9.27 ± 2.92 mm^3^ for RT+LG, and 3.94 ± 0.69 mm^3^ for RT+LNG (Figure 6F). Particularly in the RT+C‐LNG treated group, the tumor volume was only 0.51 ± 0.30 mm^3^, which was only 0.36% of the PBS control, producing a 99.64% inhibition of tumor growth. Moreover, the tumor volume in the RT+C‐LNG treated group was only 0.98% of that in RT alone, 5.47% of that in RT+LG, and 12.86% of that in the counterpart RT+LNG group, thereby verifying the striking therapeutic effects on inhibiting tumor growth. Thereafter, the whole brain tissues from each treatment were performed histological examinations (Figure 6E; Figure S27, Supporting Information), wherein the tumor lesions were denoted as cell clusters with dark nuclei. The hematoxylin‐eosin (H&E) staining images showed that the tumor lesions were hardly observable in the RT+C‐LNG treated group but obviously visualized in other groups. The quantified results indicated that the relative area of tumor lesions in the brain tissues from the RT+C‐LNG group was 6.68% of that from the RT+LNG group and 2.86% of that from the RT+LG group, thereby confirming the outstanding efficacy of the RT+C‐LNG on suppressing tumor growth (Figure 6G). Consequently, we evaluated the efficacy of RT+C‐LNG in prolonging the survival of GL261‐luc‐induced orthotopic GBM tumor models (Figure 6H). The median survival time of the orthotopic GBM tumor models was 32, 40, and 47 days for control, RT alone, and RT+Gem, respectively, but significantly extended to 60 days for RT+LG and 80 days for RT+LNG. Most encouragingly, even when the detection time was over 120 days, 5 of the 7 orthotopic GBM tumor models in the RT+C‐LNG group remained alive with a high survival rate of 71.44%, thereby confirming the distinguished benefits of RT+C‐LNG in prolonging survival time. Collectively, the combination of C‐LNG with RT produced profound inhibition of tumor growth and prolongation of survival time, thereby strikingly overcoming the RT resistance in the orthotopic GBM tumor models.

**Figure 7:**
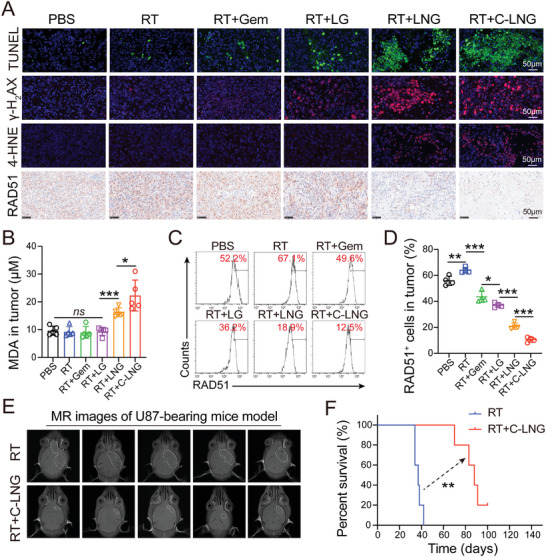
Possible mechanism of RT+C‐LNG in overcoming RT resistance. A) Cell apoptosis (TUNEL), DNA damages (γ‐H_2_AX), lipid peroxidation (4‐HNE), and DNA repair (RAD51) features in GL261‐luc induced GBM tumors from each treatment. Scale bars: 50 µm. B) The MDA content in tumor tissues from each treatment (*n* = 5). C) Typical flow cytometry of RAD51 expression in tumors from each treatment. D) Quantified RAD51 expression in tumors from each group (*n* = 5). E) MR images of U87‐induced GBM tumor models at day 30 of treatment (*n* = 5). F) Survival curves of U87‐induced GBM tumor models treated with RT and RT+C‐LNG (*n* = 5). Data are presented as mean ± SD. ^*^ *p* < 0.05, ^**^ *p* < 0.01, or ^***^ *p* < 0.001.

Considering the prominent efficacy of RT+LNG in depressing tumor growth and extending survival, we investigated their impacts on inhibiting RAD51 expression and intensifying the DNA injuries to clarify the possible mechanism of the improved therapeutic benefits (**Figure** **7**). We measured the apoptosis level in GBM tumors using terminal deoxynucleotidyl transferase dUTP nick‐end labeling assays (TUNEL) (Figure 7A). The RT+C‐LNG and RT+LNG treatments produced extensive apoptosis in the GBM tumors, as evidenced by the abundant green fluorescence signals. The immunofluorescence assays indicated the signals of γ ‐H_2_AX, a primary marker of DNA injury, were obviously detected in the RT+C‐LNG and RT+LNG treated groups, but less detectable in other groups (Figure 7A). Considering the notable spatiotemporal distribution of ONOO^−^ radicals in tumors, we examined the incidence of lipid peroxidation by measuring the products of 4‐hydroxynonenal (4‐HNE) and malondialdehyde (MDA) in tumors from different treatments (Figure 7A,B). The signals of 4‐HNE were readily detected in the RT+C‐LNG and RT+LNG treated groups, but undetected in other groups. Meanwhile, the RT+C‐LNG treatment produced higher MDA levels in tumors than other treatments (Figure 7B). These data confirmed the improved efficacy of RT+C‐LNG in inducing apoptosis, DNA injuries, and lipid peroxidation, which could be mainly ascribed to the loaded NG in the bioinspired lipoprotein system.

Regarding the essential role of RAD51 in RT resistance, we evaluated the efficacy of RT+C‐LNG treatment in downregulating RAD51 in the orthotopic GBM tumor (Figure 7C,D). The immunohistochemical analysis showed that the signals of RAD51 were barely detected in the RT+C‐LNG and RT+LNG treated tumors, but readily observed in other groups (Figure 7C). Meanwhile, the flow cytometer examinations showed that the proportion of RAD51^+^ cells in tumors treated with C‐LNG was only 18.80% of the negative control, 16.43% of the RT alone group, 28.33% of the RT+LG group, and 50.14% of the counterpart RT+LNG group (Figure 7D). These data revealed the notable effects of the RT+C‐LNG treatment in reducing RAD51 expression in GBM tumors. Subsequently, we evaluated the therapeutic benefit of C‐LNG in potentiating RT treatment in a human U87‐induced orthotopic GBM tumor model. The MRI indicated that the tumor volume in the RT+LNG treated group was much lower than that in the RT alone at day 30 of treatment (Figure 7E). Moreover, the median survival time in the RT+LNG treated group was 88 days, which was much longer than that in the RT alone (37 days) (Figure 7F), validating the effectiveness of C‐LNG in overcoming RT resistance in GBM treatments.

## 3 Conclusion

Regarding the critical role of DNA repair in RT resistance and aberrantly upregulated SR‐B1 and CCL5 in GBM tumors, we successfully synthesized a reduction‐sensitive NO donor conjugate of furoxans–gemcitabine (NG) as radio‐sensitizer and fabricated a CCL5 peptide‐modified bioinspired lipoprotein system of C‐LNG, thereby preferentially targeting the GBM tumor sites to overcome the RT resistance. Upon X‐ray radiation, the C‐LNG treatment produced abundant peroxynitrite (ONOO^−^) with considerable intratumor spatio‐temporal distribution profiles and caused notable lipid peroxidation and DNA damage. Moreover, the C‐LNG plus X‐ray radiation caused profound depression of tumor growth and extension of survival time in two orthotopic GBM tumor models. Of note, the survival rate in the RT+C‐LNG treated group remained at 71.44% at day 120 of treatment in GL261 tumors, which was superior to other treatments. Therefore, the CCL5 peptide‐modified bioinspired lipoprotein system of NG provides an encouraging platform to target the orthotopic GBM tumors and over the RT resistance for effective GBM treatment.

## Conflict of Interest

The authors declare no conflict of interest.

## Supporting information

Supporting Information

## Data Availability

The data that support the findings of this study are available in the supplementary material of this article.
