# Supplementary material for: Bioinspired Lipoproteins of Furoxans–Gemcitabine Preferentially Targets Glioblastoma and Overcomes Radiotherapy Resistance
Source: Adv Sci (Weinh). 2023 Dec 4;11(6):2306190. doi: 10.1002/advs.202306190 (PMC10853724; doi:10.1002/advs.202306190)
Supplement: Supplementary file 1 — Supporting Information [file ADVS-11-2306190-s001.pdf]

ADVANCED SCIENCE
Open Access

Supporting Information

for *Adv. Sci.*, DOI 10.1002/advs.202306190

Bioinspired Lipoproteins of Furoxans–Gemcitabine Preferentially Targets Glioblastoma and Overcomes Radiotherapy Resistance

Maoyuan Sun, Honglei Xie, Wenli Zhang, Xianlu Li, Zhan Jiang, Yiyu Liang, Guanjian Zhao, Ning Huang, Jinning Mao, Guodong Liu* and Zhiwen Zhang**

Supporting Information

Bioinspired Lipoproteins of Furoxans–Gemcitabine Preferentially Targets Glioblastoma and Overcomes Radiotherapy Resistance

Maoyuan Sun, Honglei Xie, Wenli Zhang, Xianlu Li, Zhan Jiang, Yiyu Liang, Guanjian Zhao, Ning Huang, Jinning Mao, Guodong Liu*, Zhiwen Zhang**

M. Sun, Dr. G. Zhao, Dr. N. Huang, Prof. G. Liu

Department of Neurosurgery, The Second Affiliated Hospital of Chongqing Medical University, Chongqing 400016, China. E-mail: 304678@hospital.cqmu.edu.cn

X. Li, Y. Liang, *Prof.* Z. Zhang

School of Pharmacy & Key Laboratory of Smart Drug Delivery (Ministry of Education), Fudan University, Shanghai, 201203, China. E-mail: zhangzhiwen@fudan.edu.cn

Dr. J. Mao

Health Management Center, The Second Affiliated Hospital, Chongqing Medical University, Chongqing 400016, China. E-mail: 305379@hospital.cqmu.edu.cn

Dr. H. Xie

Institute of Pharmacology, School of Pharmaceutical Sciences, Shandong First Medical University & Shandong Academy of Medical Sciences, 619 Changcheng Road, Taian 271016, China

W. Zhang

Department of Radiology, The Second Affiliated Hospital of Chongqing Medical University, Chongqing 400016, China.

Z. Jiang

Department of Oncology, The Chongqing General Hospital, Chongqing 400016, China.

Supplementary Methods

Materials: The materials of 1, 2-dipalmitoyl-sn-glycero-3-phosphocholine (DPPC), 1,2-distearoyl-sn-glycero-3-phosphoethanolamine–N-poly(ethylene glycol)2000 (DSPE–PEG), and DSPE–PEG–maleimide were provided by Sigma-Aldrich (Shanghai, China). The apolipoprotein A-1 (ApoA1) peptide (sequence, PVLDLFRELLNELLEALKQKLK), C-C motif chemokine ligand 5 (CCL5) peptide (sequence, CFPYIARPLPRAHIKEYFY), and DiIC18(5) (DiD) were purchased from Xi'an ruixi Biological Technology Co. Ltd. (Shanxi, China). NG and DSPE-PEG-CCL5 were synthesized and described in the following. The antibodies against RAD51, scavenger receptor B type 1 (SR-B1), γ-H_2AX, secondary horseradish peroxidase (HRP)-linked antibody, and 4,5-diamino-rhodamine B (DAR-1) probe were obtained from Abcam. Anti-CCL5-PE and Anti-CD31-PE were purchased from BioLegend, Inc. Glutathione (GSH), 2-(4-aminophenyl)-6-indolecarbamidine (DAPI), dihydroethidium (DHE), Alexa Fluor 488-labeled Goat Anti-Rabbit IgG(H+L), Alexa Fluor 647-labeled Goat Anti-Rabbit IgG(H+L), DNA Damage Assay Kit, terminal deoxynucleotidyl transferase dUTP nick-end labeling assays (TUNEL) assay kit, Triton X-100 and bicinchoninic acid (BCA) protein assay kit was obtained from Beyotime. 4-HNE antibody was provided by Bioss Ltd. The enzyme-linked immunosorbent assay (ELISA) Kit for the malondialdehyde (MDA) assay was ordered from Jiangsu Sumeike Biological Technology Co., Ltd. (Jiangsu, China). Peroxynitrite ($ONOO^-$) Probe was ordered from Bestbio Ltd. Anti-Nitrotyrosine was purchased from Sigma-Aldrich. BODIPY® lipid probes (C11-BODIPY) were provided by Thermo Fisher Scientific Inc. Fetel bovine serum (FBS) was purchased from Inner Mongolia Opcel Biotechnology Co., Ltd. (Inner Mongolia, China). D-luciferin sodium was purchased from Yuanye Bio-Technology Co. Ltd. (Shanghai, China).

Cell culture. The murine GL261 cells and the human U87 cells were provided by the Cell Bank of Shanghai, Chinese Academy of Sciences (CAS, Shanghai, China). The GL261 cells with

stable expression of luciferase were provided by iCell Bioscience Inc (Shanghai, China). Cells were cultured with high glucose Dulbecco's modified eagle medium (DMEM) (Hyclone) containing 10% FBS (Inner Mongolia Opcel) and 1% penicillin-streptomycin (Hyclone) in a humidified atmosphere that contained 5% CO_2 at 37 °C.

Animals. Male C57BL/6J mice (6 - 8 weeks) and male BALB/c nude mice (6 - 8 weeks) were purchased by Enswell Biotechnology Ltd (Chongqing, China). The animal experiments were performed in the Chongqing Medical University Laboratory Animal Center and the protocols were approved by the Animal Ethics Committee of the Chongqing Medical University (Approved No. IACUC-CQMU-2023-0096). To establish the orthotopic GBM model, GL261/GL261-luc (3×10^5/μL, 5 μL), or U87 cells (1×10^5/μL, 5 μL) were inoculated into the right striatum (lateral: 1.8 mm, longitudinal: 0.6 mm, depth: 2.5 mm). Bioluminescence imaging and magnetic resonance imaging (MRI) were used to monitor the tumor size.

Bioinformatic analysis of GBM patients. The expression of RAD51, SR-B1, and CCL5 in GBM patients (n = 153) and healthy people (n = 1157) was assessed in TCGA and GTEx databases using visualization tools in Sangerbox 3.0 (http://sangerbox.com/home.html). The data were transformed as $\log_2$ (TPM + 1) and n is the number of biologically independent samples. The effect of RAD51 expression level on the survival benefits of GBM patients on the placebo arm of the AVAglio dataset was analyzed based on publicly available data (GSE84010) from GEO datasets. Furthermore, the effect of RAD51 expression level on the survival benefits of GBM patients with radiotherapy was analyzed based on publicly available data (GSE186057) from GEO datasets (https://www.ncbi.nlm.nih.gov/geo). Survival curves of GBM patients were generated by an R language package and presented with Kaplan-Meier plots.

Human tumor specimens: The tumor and peritumor tissues of GBM patients were obtained from the Department of Neurosurgery at the Second Affiliated Hospital of Chongqing Medical University. All specimens were obtained with written informed consent and standard research protocol approved by the Ethics Committee of the Second Affiliated Hospital of Chongqing Medical University (Approved No. YLS 2023-079).

Immunofluorescence assays of tumor and peritumor tissues from GBM patients. The tumor and peritumor tissues from GBM patients were collected under routine surgical resections. To detect the expression of RAD51, sections were treated with Fixation/Permeabilization solution (BD Cytofix/Cytoperm), stained with primary antibodies of RAD51 (Abcam, ab133534, 1:200), and followed by Alexa Fluor® 647 antibody (Beyotime, A0468, 1:200). In contrast, the sections were counterstained with DAPI for visualization under a confocal laser scanning microscope (CLSM, LSM710. Carl Zeiss, Germany). The expression of indicators exhibited from the confocal image was further analyzed by Image J software.

To detect the expression of SR-B1, the sections were fixed and permeable with Fixation/Permeabilization solution (BD Cytofix/Cytoperm), then stained with primary antibodies of SR-B1 (Abcam, ab217318, 1:100) and followed by Alexa Fluor® 647 antibody (Beyotime, A0468, 1:200). In contrast, the sections were counterstained with DAPI for visualization under CLSM (LSM710. Carl Zeiss, Germany). The images were further analyzed by Image J software.

To detect the expression of CCL5, the sections were treated with Fixation/Permeabilization solution (BD Cytofix/Cytoperm), then stained with Anti-CCL5-PE (BioLegend, 515503, 1:200). In contrast, the sections were counterstained with DAPI for visualization under CLSM (LSM710. Carl Zeiss, Germany). The images were further analyzed by Image J software.

Preparation and characterization of C-LNG. C-LNG was fabricated with DPPC, DSPE-PEG-CCL5, NG, and ApoA1 peptide. In brief, DPPC, DSPE-PEG-CCL5, and NG (6: 3: 1, w/w) were accurately weighed, dissolved in methanol, evaporated to form a thin film in a round flask, dispersed with purified water, and sonicated with a probe for 1 min. Then, 1 mg of ApoA1 peptide was added to the mixed solution and performed three heat-cooling cycles between 50 °C and 4 °C to obtain C-LNG. In contrast, the counterpart formulation of LG (DPPC, DSPE-PEG, and ^{14}C-Gem, 6: 3: 1, w/w) and LNG (DPPC, DSPE-PEG, and NG, 6: 3: 1, w/w) were fabricated in the same preparation process.

The morphology of LG, LNG, and C-LNG was measured using transmission electron microscopy (TEM) (Tecnai G2 S-Twin, FEI) after staining with saturated uranyl acetate solution. The particle size distribution of LG, LNG, and C-LNG was measured using a dynamic light scattering (DLS) analysis on a Nano ZS 90 Zetasizer (Malvern, UK). The encapsulation efficiency (EE) of ^{14}C-Gem in LG, NG in LNG, and NG in C-LNG was respectively determined using high-performance liquid chromatography (HPLC) analysis (Agilent, 1290II-6460) under the following condition: mobile phase, acetonitrile-0.1% formic acid water (45/55, v/v); column, BEH-C18 1.7 μm (2.1×100mm); flow rate, 0.3 mL /min; detect wavelength: 269 nm. The unloaded drug was separated from the formulations using ultrafiltration tubes (30 KD, Vivaspin500, Sartorius) by centrifuging at 4 °C for 10 min. The drug amount was determined by the HPLC method to calculate the EE and drug loading capacity in the bioinspired lipoprotein system. To further evaluate their stability in the physiological fluids, these formulations were incubated in phosphate buffer solution (PBS) (pH 7.4) and PBS (pH 7.4) plus 10% FBS for 48 h, and measured by DLS (Malvern, UK). Moreover, C-LNG was incubated in the PBS (pH 7.4) and PBS (pH 7.4)+10% FBS at 37 °C for 48 h. At predetermined time points, the EE values of NG from C-LNG was monitored by the aforementioned HPLC method to evaluate their stability.

To investigate the reduction-sensitive properties, these formulations were respectively incubated in PBS (pH 7.4) +10 mM GSH at 37 °C. The morphologies of LG, LNG, and C-LNG were detected by TEM, and the particle size distribution was measured by DLS analysis at 48 h of incubation. Moreover, the degraded products were analyzed by liquid chromatography-electrospray ionization mass spectrometry (Agilent Technologies, Inc. EP-C18). To determine the time-dependent drug release profiles, C-LNG was incubated in PBS (pH 7.4) +10 mM GSH for 48 h at 37 °C. At predetermined time point, the NG amount remained in the C-LNG was quantified by the aforementioned HPLC method to calculate the percentage of drug release. To verify the NO release from these formulations, they were incubated in PBS (pH 7.4) + 10 mM GSH at 37 °C with a NO fluorescence probe of DAR-1 (Abcam, ab145388, 20 μM). At predetermined time intervals, samples were analyzed using a microplate reader (Ex560nm, Em595nm) (Bio-Tek Instrument Inc., USA).

NO production in GL261 cancer cells. The NO production in GL261 cells was examined by CLSM and flow cytometry analysis (BD FACS Vantage SE, USA). In brief, GL261 cells (1×10^5 cells per well) were seeded into the confocal dish or 6-well plate and cultured overnight. Cells were pretreated with a NO fluorescence probe of diaminofluorescein-FM diacetate (DAF-FM DA, Beyotime, 1:1000) for 1 h at 37 °C, then replaced with fresh culture media containing LG, LNG, and C-LNG at 20 μg/mL of NG or comparable concentrations for a further 24 h of incubation. Afterward, cells from each treatment were harvested for CLSM detections and flow cytometry examinations.

To detect their impact on cellular GSH in GL261 cells, cells were seeded into a 6-well plate (1×10^5 cells per well), cultured for 12 h, and incubated with LG, LNG, and C-LNG at 20 μg/mL of NG or comparable concentrations for 24 h. The intracellular level of GSH was quantified according to the protocol of a GSH assay kit (Beyotime, S0052).

$ONOO^-$ production and characterization of lipid peroxidation. Regarding the efficient NO generation from LNG and C-LNG groups *in vitro*, we further measured the production of $ONOO^-$ upon X-ray radiation. GL261 cells (1×10^5 cells per well) were seeded into the confocal dish and cultured overnight. Then, they were incubated with free Gem, LG, LNG, and C-LNG at 20 μg/mL of NG or comparable concentrations. After 4 h of incubation, cells from each treatment were exposed to X-ray radiation at 2 Gy (6 MV, Varian, VitalBeam, USA). Then, cells were fixed with 4% paraformaldehyde, and incubated with anti-nitrotyrosine (Sigma-Aldrich, 1:200) overnight at 4 °C followed by Alexa Fluor 647-labeled IgG (H+L) (Beyotime, 1:200) for 1 h at room temperature, and then counterstained with DAPI for visualization under CLSM. Moreover, the production of $ONOO^-$ was further detected and quantified using a BBoxiProbe® O^{58} fluorescent dye on a microplate reader (Ex 516nm, Em 606nm, Bio-Tek Instrument Inc., USA)

To identify their impact on the lipid peroxidation induced by $ONOO^-$, GL261 cells (1×10^5 cells per well) were seeded into a 6-well plate and cultured for 12 h. Then, they were respectively incubated with free Gem, LG, LNG, and C-LNG at 20 μg/mL of NG or comparable concentrations. After 4 h, cells were exposed to X-ray radiation at 2 Gy (6 MV, Varian, VitalBeam, USA) and incubated for a further 6 h. Cells were stained with C11-BODIPY$^{581/591}$ (5 μM) for 15 minutes at room temperature and then analyzed by flow cytometry (BD FACSVantage SE, USA). Meanwhile, the generation of lipid peroxidation was observed by CLSM. In brief, the GL261 cells (1×10^5 cells per well) were seeded into the confocal dish, cultured for 12 h, and performed with the same process as above. The production of lipid peroxides in the cell membrane was visualized by CLSM, wherein the oxidized lipid was denoted as green fluorescence signals.

DNA damage and repair. The impact of C-LNG mediated radiation treatment on DNA damage was performed using DNA Damage Assay Kit (γ-H_2AX Immunofluorescence, Beyotime).

GL261 cells (1×10^5 cells per well) were seeded into the confocal dish and cultured overnight. Then, cells were respectively treated with free Gem, LG, LNG, and C-LNG at 20 μg/mL of NG or comparable concentrations for 4 h, exposed to X-ray radiation at 2 Gy, and incubated for a further 20 h. Cells were treated with the Fixation/Permeabilization solution, then stained with primary γ-H_2AX antibody following the manufacturer's protocol (DNA Damage Assay Kit, C2035S, Beyotime). Afterward, cells were stained with DAPI for visualization under CLSM.

Considering the essential role of RAD51 in DNA repair, we measured the RAD51 expression in cells from each treatment by immunofluorescence assays. In brief, GL261 cells (1×10^5 cells per well) were seeded into the confocal dish and followed the same treatment as above. Cells were treated with the Fixation/Permeabilization solution, stained with primary RAD51 antibody (Abcam, ab133534, 1:200) and Alexa Fluor 647-labeled IgG (H+L) (Beyotime, A0468, 1:200), then counterstained with DAPI for visualization under CLSM.

Then, the expression of the γ-H_2AX and RAD51 in cells from each treatment was further investigated by western blot analysis. In brief, GL261 cells were seeded into the 6-well plate (5×10^5 cells per well) and cultured overnight. Then, cells were respectively treated with free Gem, LG, LNG, and C-LNG at 20 μg/mL of NG or comparable concentrations for 4 h, exposed to X-ray radiation at 2 Gy, and incubated for a further 20 h. Afterward, cells from each treatment were collected and treated with the pre-cooled RIPA lysis buffer (Beyotime, China) and centrifuged at 12,000 rpm at 4 °C for 20 minutes. The protein concentration in each sample was quantified with a BCA protein assay kit. The expression of γ-H_2AX and RAD51 was measured using a primary γ-H_2AX antibody (Abcam, ab81299, 1: 500) and a primary RAD51 antibody (Abcam, ab133534, 1:1000), followed by a secondary HRP-linked antibody (Abcam, ab97051, 1: 2000) according to the manufacturer's protocols for detections (Bio-Rad, USA).

In vitro therapeutic efficacy evaluations.

The cytotoxicity of C-LNG was measured in GL261 cells and Bend.3 cells by a cell counting kit (CCK-8). Both of them were seeded into the 96-well plate (1×10^4 cells per well) and cultured overnight, and then incubated with C-LNG at NG concentration ranging from 0 to 400 ng/mL for 20 h. The cell viability from each treatment was monitored using the CCK-8 kit for quantification. To evaluate the therapeutic efficacy of different treatment strategies in vitro, GL261 cells were seeded into the 96-well plate (1×10^4 cells per well) and cultured overnight. Then, they were respectively treated with free Gem, LG, LNG, and C-LNG at 20 μg/mL of NG or comparable concentrations for 4 h, exposed to X-ray radiation at 2 Gy, and incubated for a further 20 h. The cell viability from each treatment was measured using the CCK-8 assay kit on a microplate reader.

Next, the therapeutic efficacy was further measured by colony formation assays. In brief, GL261 cells were seeded into the 6-well plate (5×10^3 cells per well) and cultured overnight. Then, they were respectively treated with free Gem, LG, LNG, and C-LNG at 20 μg/mL of NG or comparable concentrations for 4 h, exposed to X-ray radiation at 2 Gy, and further cultured for 10 days to form clusters of cells. Afterward, the cell colonies were fixed with 4% paraformaldehyde and stained with Giemsa dye.

Targeting behavior to orthotopic GBM tumors. To evaluate their targeting to the GL261-luc-induced orthotopic GBM tumors, both LNG and C-LNG were fluorescently labeled with DiD for the imaging. The formation of the GL261-luc-induced orthotopic GBM tumor model was verified by bioluminescence assays prior to the experiments. At different time intervals after injection (1.0 mg/kg of DiD), the fluorescence signals from each group were monitored using an IVIS imaging system (IVIS, Perkin Elmer, U.K.), and analyzed by corresponding software. After 12 h post-injection, mice were sacrificed, and the major organs including the heart, liver, spleen, lung, kidney, and brain were collected for visualization under the imaging system. To detect their access to the orthotopic tumor lesions, the brain tissues with GBM tumors were

frozen-sectioned at 10 μm and counterstained with anti-CD31-PE (BioLegend, 1: 200) and DAPI for visualization under CLSM. Then, the gemcitabine amount in the major organs from LNG and C-LNG treated groups were quantified by liquid chromatography-mass spectrometry (LC-MS, Agilent Technologies, Inc. EP-C18). At 12 h after i.v. injection, the major organs including heart, liver, spleen, lung, kindey, normal brain tisses and GBM tumor regions were carefully collected, weighed, and homogenized with 1% Triton X-100 for further quantification. To detect the in vivo pharmacokinetic profiles, the DiD-labeled LNG and C-LNG were injected into healthy mice via tail vein, and the blood samples were collected at certain time intervals. The fluorescence intensity in these blood samples were quanfied to calculate the elimination half-life of LNG and C-LNG.

In vivo NO production and spatio-temporal distribution of $ONOO^-$. To detect the NO production and its distribution profiles in the orthotopic GBM tumor tissues, the tumor models were respectively injected with PBS, free Gem, LG, LNG, and C-LNG at 5.0 mg/kg of NG (or comparable doses) via the tail vein. At 12 h of injection, the tumor tissues were harvested, sectioned at 10 μm (Leica 1950, Germany), and then stained with a NO fluorescence probe of DAR-1 (NO probe, 100 μM) for visualization under CLSM.

Given the efficient NO production in orthotopic GBM tumors, we evaluated the spatio-temporal distribution profiles of $ONOO^-$. The orthotopic GBM tumor models were respectively injected with PBS, free Gem, LG, LNG, and C-LNG at 5.0 mg/kg of NG (or comparable doses) via the tail vein. At 12 h of injection, the brain tissues were exposed to X-ray radiation (2 Gy). To detect the ROS production, the tumor models were injected with 100 μL of DHE (10 μM) via the tail vein 30 minutes prior to the X-ray radiation. Then, the tumor tissues in the brain were harvested, sectioned at 10 μm, and counterstained with DAPI for visualization under CLSM. To detect the $ONOO^-$ production in the tumor regions, the tumor sections were treated with Fixation/Permeabilization solution, stained with anti-Nitrotyrosine (Sigma-Aldrich,

N0409, 1:100) overnight at 4 °C and Alexa Fluor 647-labeled IgG (H+L) (Beyotime, A0468, 1:200) for 1 h at room temperature, and counterstained with DAPI to monitor the $ONOO^-$ signals under CLSM.

Then, the time-dependent distribution profiles of ROS from the LG-treated tumors with radiation (RT+LG) and $ONOO^-$ radicals from the C-LNG treated tumors with radiation (RT+C-LNG) were measured by CLSM. In the RT+LG groups, 100 μL of DHE (10 μM) was injected via the tail vein 30 minutes prior to the X-ray radiation (2 Gy). Then, the tumor tissues were collected at 0 h, 2 h, 4 h, 12 h, and 24 h of radiation, sectioned 10 μm, counterstained with DAPI, and visualized under CLSM to monitor the fluorescence signals of ROS. In the RT+C-LNG groups, the tumor tissue was collected at 0 h, 2 h, 4 h, 12 h, and 24 h after X-ray radiation and sectioned 10 μm. These sections were treated with Fixation/Permeabilization solution, stained with anti-Nitrotyrosine (Sigma-Aldrich, N0409, 1:100) overnight at 4 °C and Alexa Fluor 647-labeled IgG (H+L) (Beyotime, A0468, 1:200) for 1 h at room temperature. Afterward, the tumor sections were counterstained with DAPI for visualization under CLSM to record the fluorescence signals of $ONOO^-$. The fluorescence signals of ROS and $ONOO^-$ were analyzed by the Image J software.

In vivo therapeutic efficacy of RT+C-LNG in orthotopic GBM tumor model. To evaluate the tumor inhibition efficacy, the GL261-luc-induced orthotopic GBM tumor models were respectively treated with PBS, RT, RT+Gem, RT+LG, RT+LNG, and RT+C-LNG on day 10 of tumor inoculation at 5 mg/kg of NG or a comparable dose. After 12 h post-injection, the mice head was exposed to X-ray radiation at 2 Gy (6 MV, Varian, VitalBeam, USA). The bioluminescence signals of the brain tissues were recorded by the IVIS imaging system on days 10, 20, and 30 to monitor the progression profiles. Moreover, MRI was used to monitor the tumor size on day 30 (Maximum length diameter×Maximum width diameter×Height×3.14/6). Moreover, the survival rate from each treatment was monitored until 120 days of treatment to

calculate the median survival time. At day 30 of treatment, 4 mice from each treatment were autopsied, and the major organs and the blood samples from each treatment were collected for further measurements. The brain tissues with GBM tumor lesions were measured by hematoxylin and eosin (H&E) staining method to monitor the tumor lesions. Other major organs including the heart, liver, spleen, lung, and kidney were measured by the HE staining method to evaluate the biosafety. Meanwhile, the blood samples were conducted in the hematology and blood biochemical tests.

To clarify the possible mechanism of the enhanced efficacy, the intracranial GBM mice were divided into six groups on day 10 of tumor inoculation and treated as described above. On day 18, the tumors from brain tissues were collected and sectioned at 10 μm for further measurements. To detect the apoptosis level, these sections were stained with a TUNEL assay kit to evaluate the apoptosis in tumor tissue (Beyotime, C1086). To detect the lipid peroxidation, the tumor sections from each treatment were labeled with 4-HNE antibody (Bioss,6313R, 1:200) and followed stain with Alexa Fluor 647-labeled IgG (H+L) (Beyotime, A0468, 1:200) for visualization. Meanwhile, the levels of MDA in tumor tissues were also determined by the Assay kits according to the manufacturer's protocols (Sumeike). To measure the DNA damages, the sections were stained with γ-H_2AX antibody (Abcam, ab81299, 1: 200) and Alexa Fluor 647-labeled IgG (H+L) (Beyotime, A0468, 1:500) for visualization. To determine the DNA repair capacity, immunohistochemistry (IHC) analysis was performed by labeling with RAD51 antibody (Abcam, ab133534, 1:100). Meanwhile, the tumor tissues were homogenized into a single-cell suspension. Cells were treated with Fixation/Permeabilization solution, then stained with anti-RAD51 antibody (Abcam, ab133534, 1:200) and Alexa Fluor 488-labeled Goat Anti-Rabbit IgG (H+L) (Beyotime, A0468, 1:500) for flow cytometer examinations to quantify the percentage of RAD51-positive cells.

Tumor inhibition study in U87 model. The intracranial human GBM model was established with U87 cells in male BALB/c nude mice (6 - 8 weeks). In brief, U87 cells (1×10^5/μL, 5 μL) were inoculated into the right striatum of mice (lateral: 1.8 mm, longitudinal: 0.6 mm, depth: 2.5 mm). The U87-induced GBM tumor models were respectively treated with RT alone and RT+C-LNG group. On days 10, 12, 14, and 16 of tumor inoculation, mice were injected with PBS and C-LNG at 5 mg/kg of NG. After 12 h post-injection, the mice head was exposed to X-ray radiation at 2 Gy (6 MV, Varian, VitalBeam, USA). On day 30, the tumor volume was evaluated using MRI. The survival of mice from each treatment was recorded to calculate the median survival time.

Statistical analysis. Statistical analysis was performed with GraphPad Prism 9.0.0 software and data were presented as means ± SD. The results were analyzed by two-tailed Student's t-tests in variable groups. The survival analyses were conducted using the Kaplan–Meier curves and the log-rank test. Significant differences are indicated as $^{*}P < 0.05$, $^{**}P < 0.01$, or $^{***}P < 0.001$.

Supplementary Results

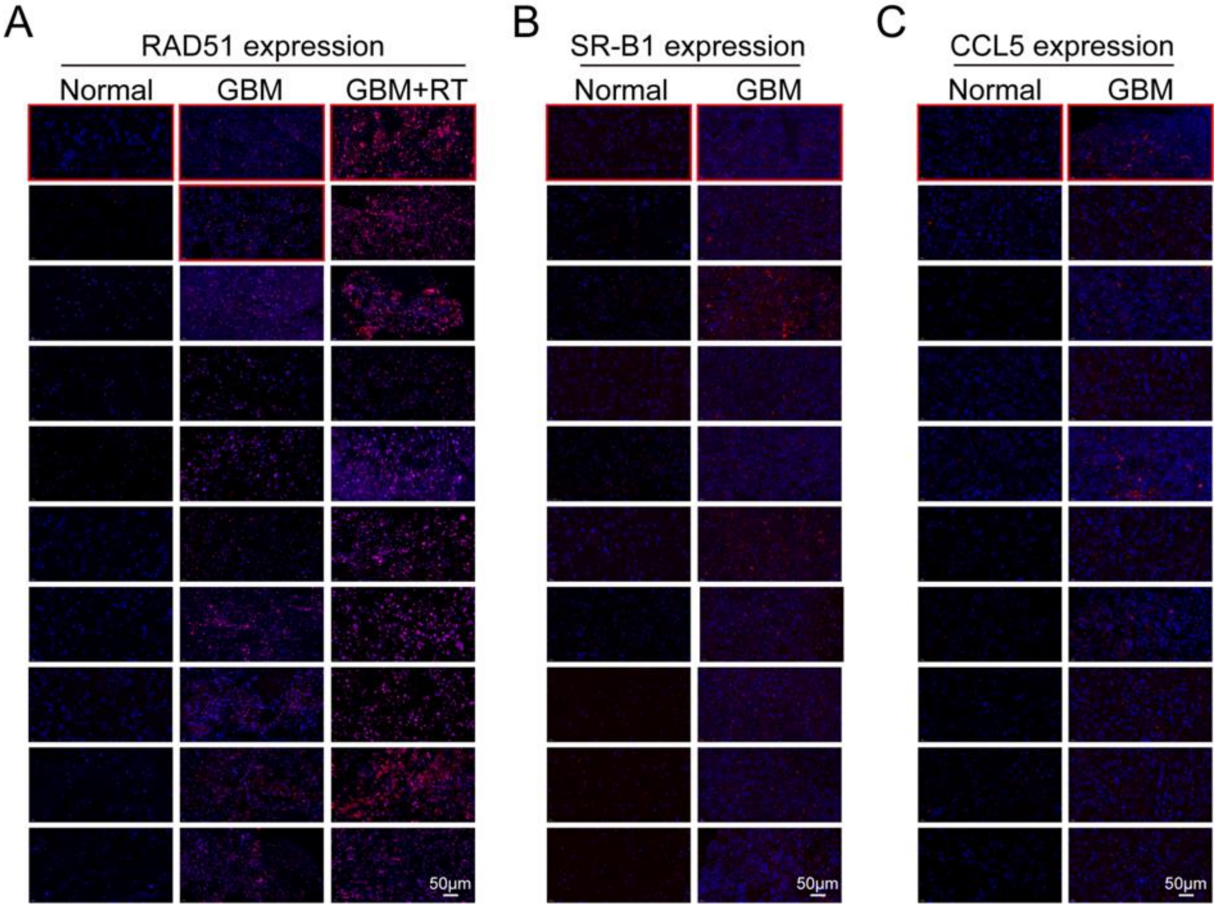

Figure S1 Immunofluorescence assays of (A) RAD51, (B) SR-B1, and (C) CCL5 in normal brain tissues and tumor tissues resected from GBM patients, or tumor tissues resected from GBM patients with radiotherapy. (n = 10). Scale bar: 50 μm.

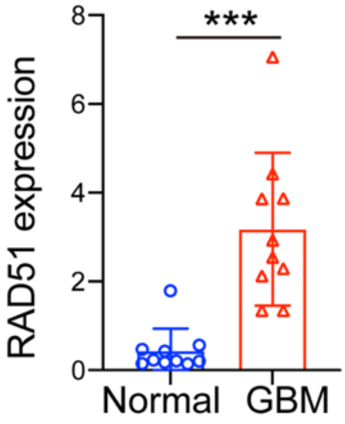

Figure S2 Quantified expression of RAD51 in normal brain tissues and tumor tissues resected from GBM patients (n = 10). Scale bar: 50 μm. $^{***}P < 0.001$.

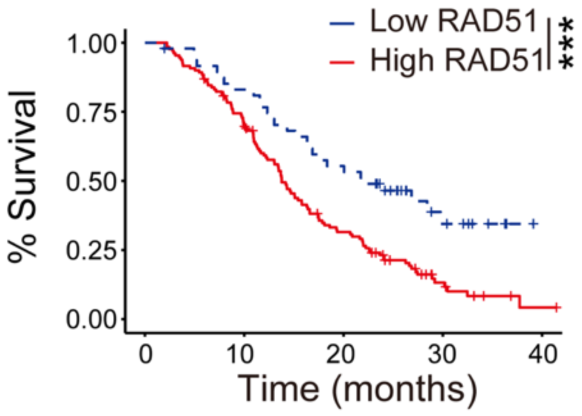

Figure S3 Survival analysis of GBM patients on the placebo arm of the AVAglio dataset with high (n = 130) and low (n = 48) RAD51 expression. $^{***}P < 0.001$.

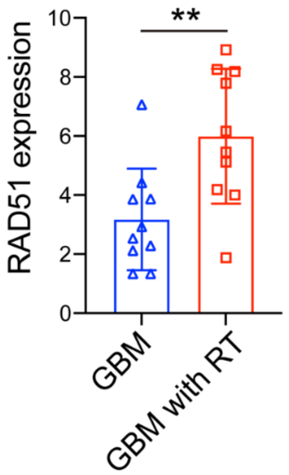

Figure S4 Quantified expression of RAD51 in tumor tissue resected from GBM patients with or without radiotherapy (n = 10). Scale bar: 50 μm. $^{***}P < 0.001$.

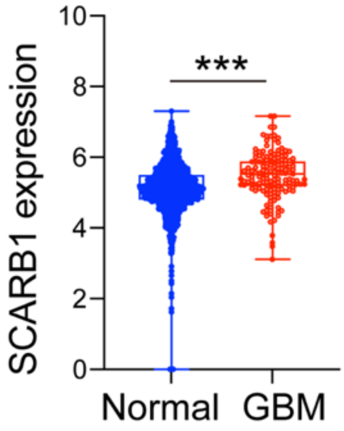

Figure S5 Boxplot of SR-B1 (encoded by the SCARB1 gene) expression in GBM patients (n = 153) and healthy people (n = 1157) based on TCGA and GTEx databases. The data were transformed as $\log_2$(TPM + 1). $^{***}P < 0.001$.

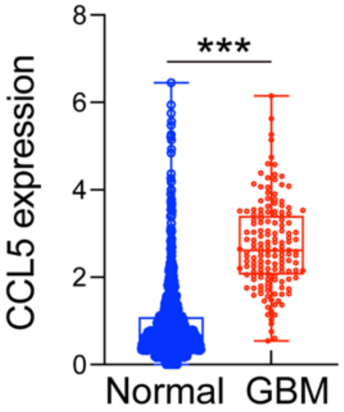

Figure S6 Boxplot of CCL5 expression in GBM patients (n = 153) and healthy people (n = 1157) based on TCGA and GTEx databases. The data were transformed as $\log_2$(TPM + 1). $^{***}P < 0.001$.

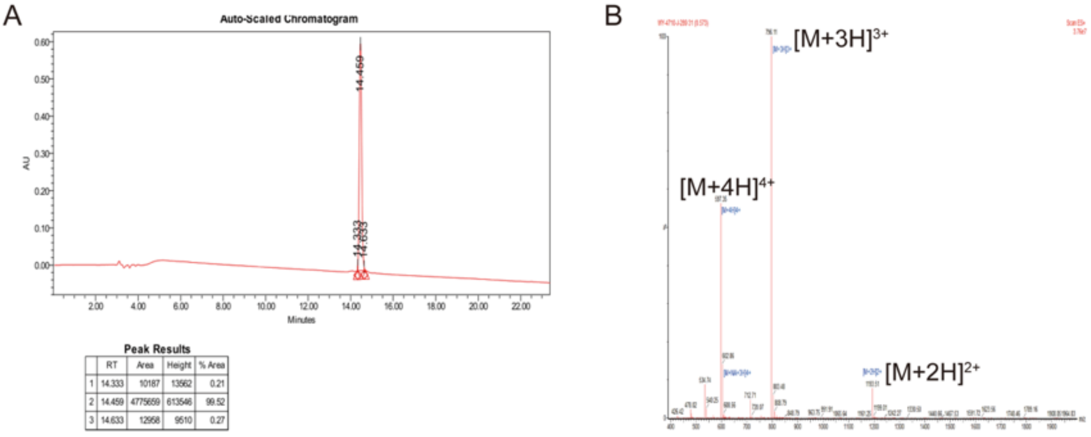

	RT	Area	Height	% Area
1	14.333	10187	13562	0.21
2	14.459	4775659	613546	99.52
3	14.633	12958	9510	0.27

Figure S7 HPLC (A) and MS (B) characterizations of CCL5 peptide (sequence, CFPYIARPLPRAHIKEYFY): Purity 99.52%.

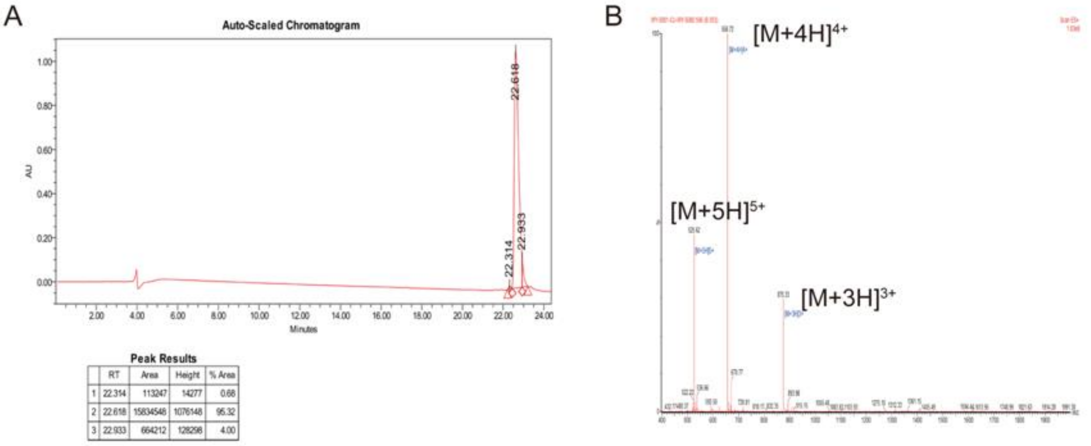

	RT	Area	Height	% Area
1	22.314	113247	14277	0.68
2	22.618	15834548	1076148	95.32
3	22.933	664212	128298	4.00

Figure S8 HPLC (A) and MS (B) characterizations of ApoA1 peptide (sequence, PVLDLFRELLNELLEALKQKLK): Purity 95.32%.

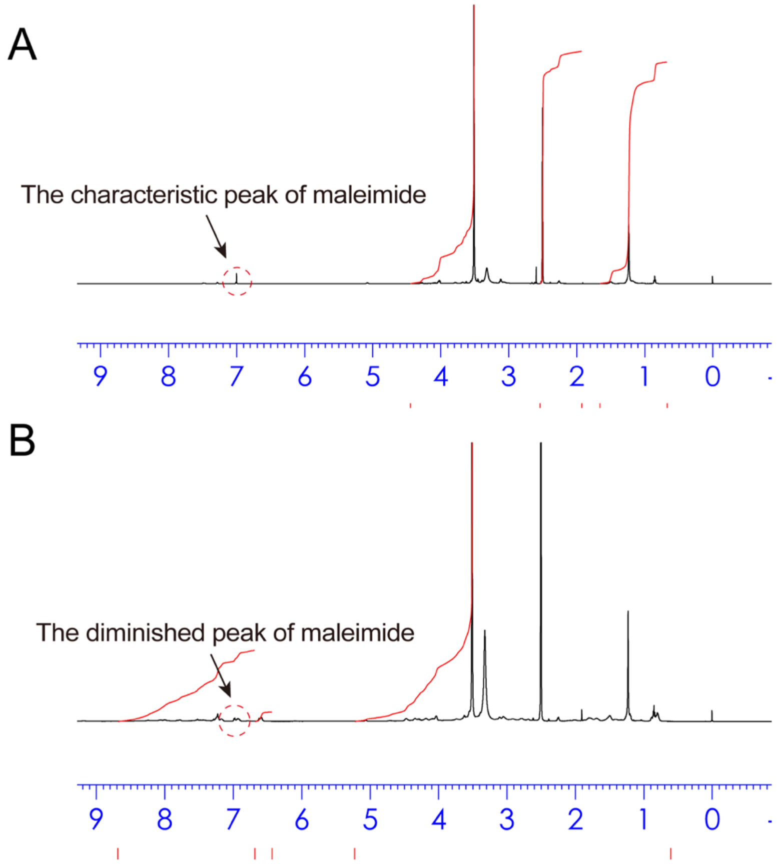

Figure S9 Synthesis and characterization of DSPE-PEG-CCL5. DSPE-PEG-CCL5 was synthesized by conjugating the thiol group of CCL5 peptides (CFPYIARPLPRAHIKEYFY) to DSPE-PEG-maleimide. In brief, 20.0 mg of CCL5 peptides and 24.26 mg of DSPE-PEG-maleimide were dissolved in 1 mL DMF with 5 μL of triethylamine (TEA) and then stirred at room temperature overnight. Afterward, the reaction solution was purified with a dialysis method (molecular weight of cut off: 3500 Da) for 36 h, and then freezing dried to get a white solid powder. The formation of DSPE-PEG-CCL5 was verified by the ^{1}H-NMR spectrum. (A) The characteristic peak of the maleimide of DSPE-PEG-maleimide was marked. (B) The characteristic peak of maleimide was diminished after conjugating the thiol group of the CCL5 peptides, suggesting the successful synthesis of DSPE-PEG-CCL5.

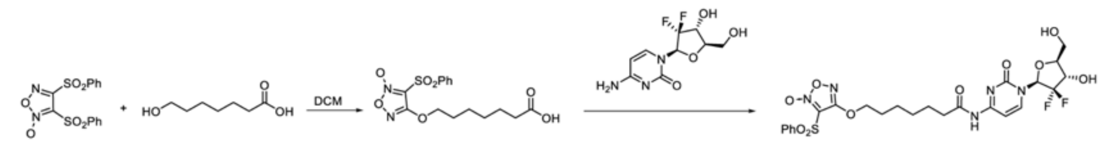

Compound 1 Compound 2 Compound 3 Compound NG

Figure S10 Synthesis procedure (two steps) and characterizations of NG.

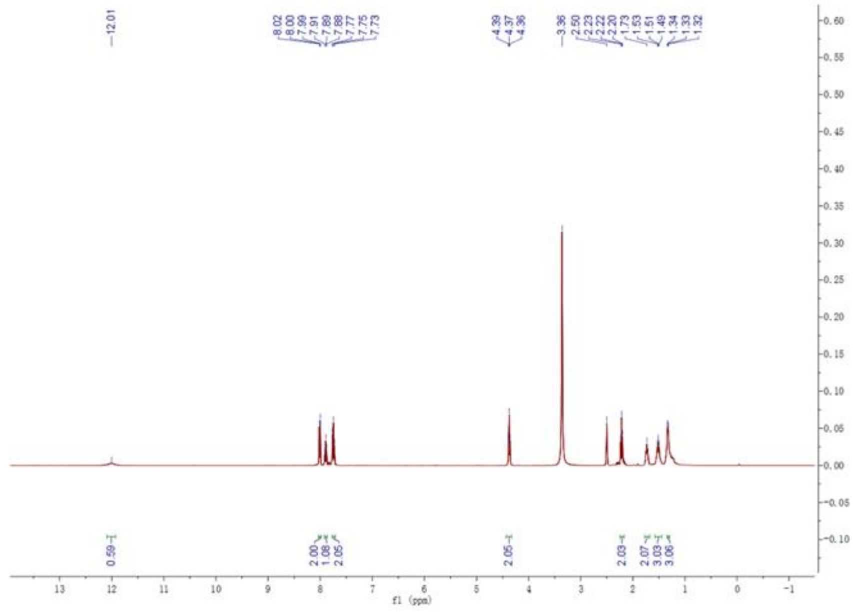

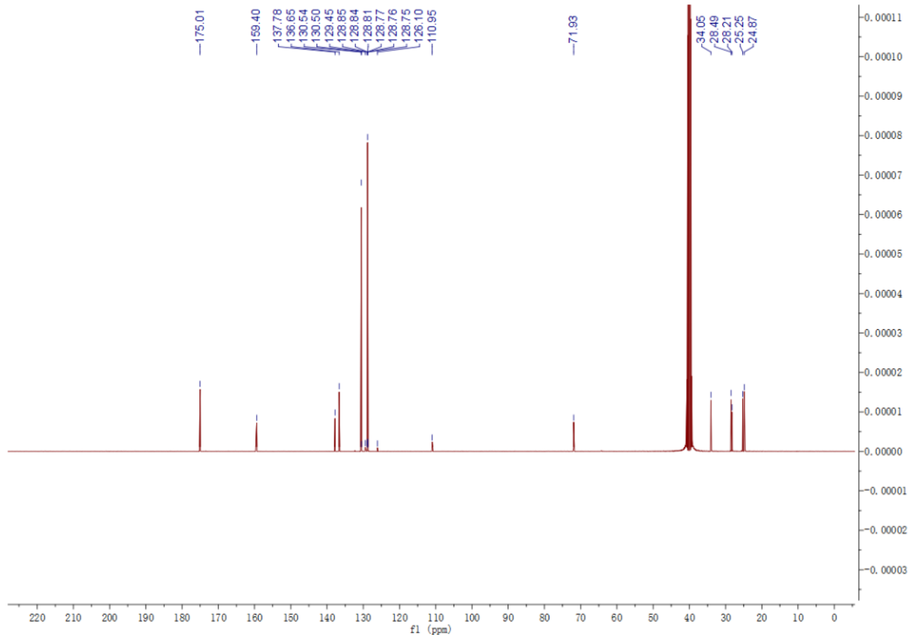

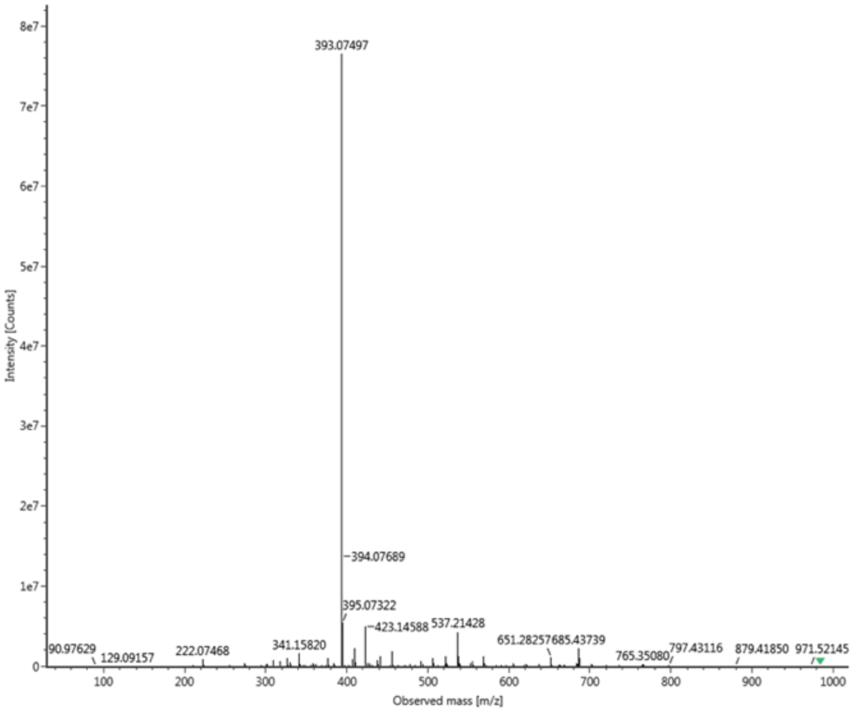

Compound 3: The 3,4-bis(phenylsulfonyl)-1,2,5-oxadiazole 2-oxide 5.0 g and 2.0 g of 7-hydroxyheptanoic acid were dissolved in 30 mL of dichloromethane (DCM) and then mixed with1,8-diazabicyclo [5,4,0] undec-7-ene (DBU) 4.1 g. The mixture was stirred at 25 °C for 8 h and the reaction solution was concentrated under reduced pressure to yield a yellow liquid, and then was extracted with ethyl acetate and saturated brine. Afterward, the combined organic layer was concentrated, and then purified on a silica gel column with eluted ethyl acetate/ petroleum ether (10:1, v/v) to give the desired product of 2.8 g (yield, 54%). which was characterized by the NMR spectrum. ^{1}H NMR (400 MHz, DMSO-d6) δ (ppm): 12.01 (s, 1H), 8.02 – 7.99 (t, *J* = 3 Hz, 2H), 7.91 – 7.89 (t, *J* = 1.9 Hz, 1H), 7.77 – 7.73 (t, *J* = 3.9 Hz, 2H), 4.39 – 4.36 (d, *J* = 3 Hz, 2H), 2.23– 2.20 (t, *J* = 3 Hz, 2H), 1.73 – 1.72 (d, *J* = 1.5 Hz, 2H), 1.53 – 1.49 (m, *J* = 3 Hz, 3H), 1.34 – 1.32 (m, *J* = 1.5 Hz, 3H). ^{13}C NMR (101 MHz) δ (ppm): 175.01, 159.40, 137.78, 136.65, 130.54, 130.50, 129.45, 128.85, 128.84, 128.81, 128.77, 128.76, 128.75, 126.10, 110.95, 71.93, 34.05, 28.49, 28.21, 25.25, 24.87. HRMS calcd for C15H18N2O7S, $[M+Na]^{+}$393.07324; found 393.07497.

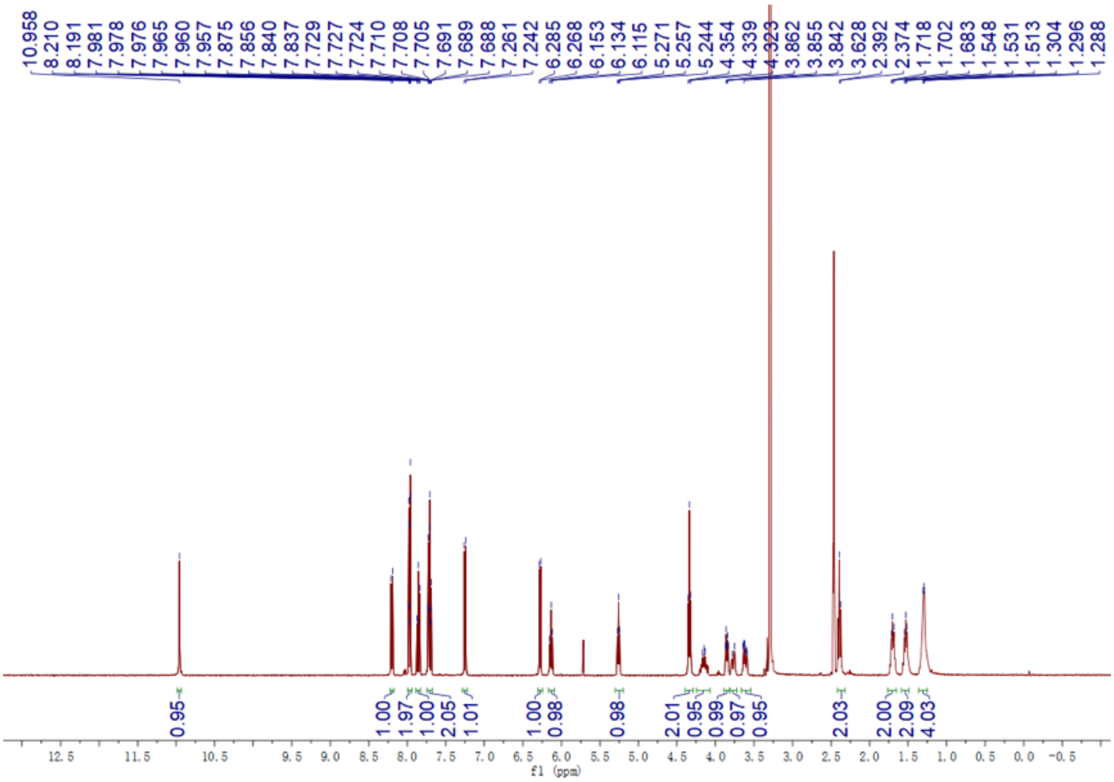
10.958
8.210
8.191
7.981
7.978
7.976
7.965
7.960
7.957
7.875
7.856
7.840
7.837
7.729
7.727
7.724
7.710
7.708
7.705
7.691
7.689
7.688
7.261
7.242
6.285
6.268
6.153
6.134
6.115
5.271
5.257
5.244
4.354
4.339
4.323
3.862
3.855
3.842
3.628
2.392
2.374
1.718
1.702
1.683
1.548
1.531
1.513
1.304
1.296
1.288
0.95
1.00
1.97
1.00
2.05
1.01
1.00
0.98
0.98
2.01
0.95
0.99
0.97
0.95
2.03
2.00
2.09
4.03
f1 (ppm)

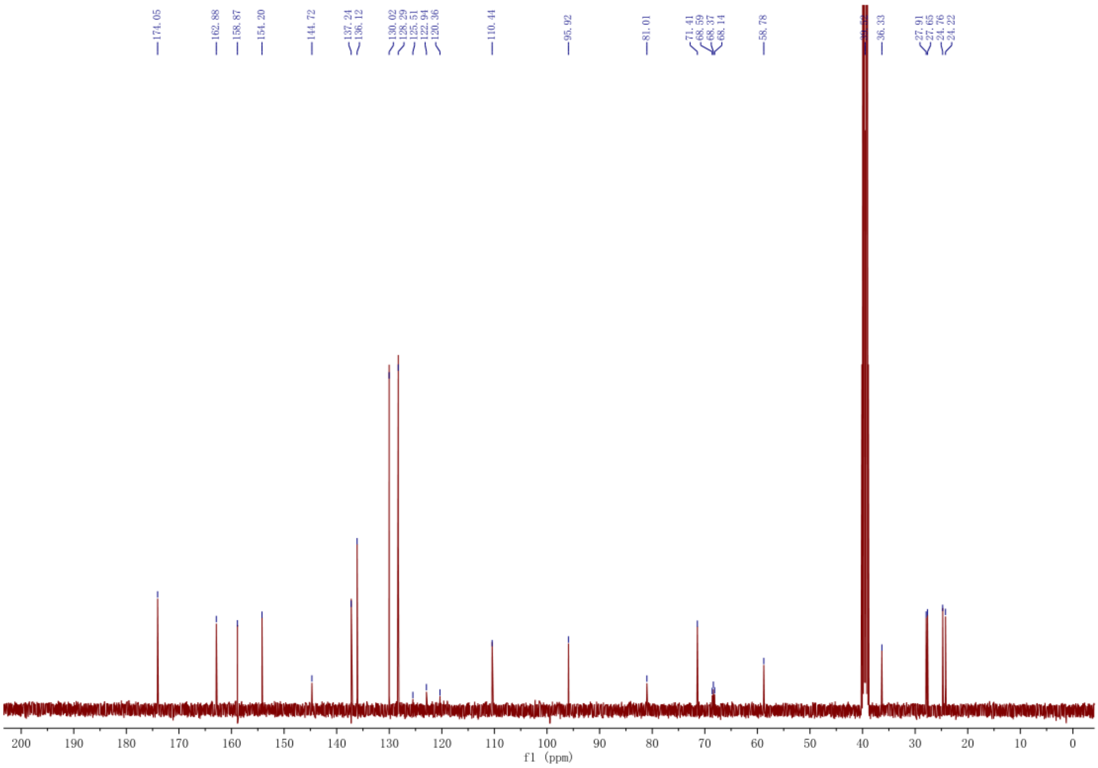
174.05
162.88
158.87
154.20
144.72
137.24
136.12
130.02
128.29
125.51
122.94
120.36
110.44
95.92
81.01
71.41
68.39
68.37
68.14
58.78
36.33
27.91
27.65
24.76
24.22
f1 (ppm)

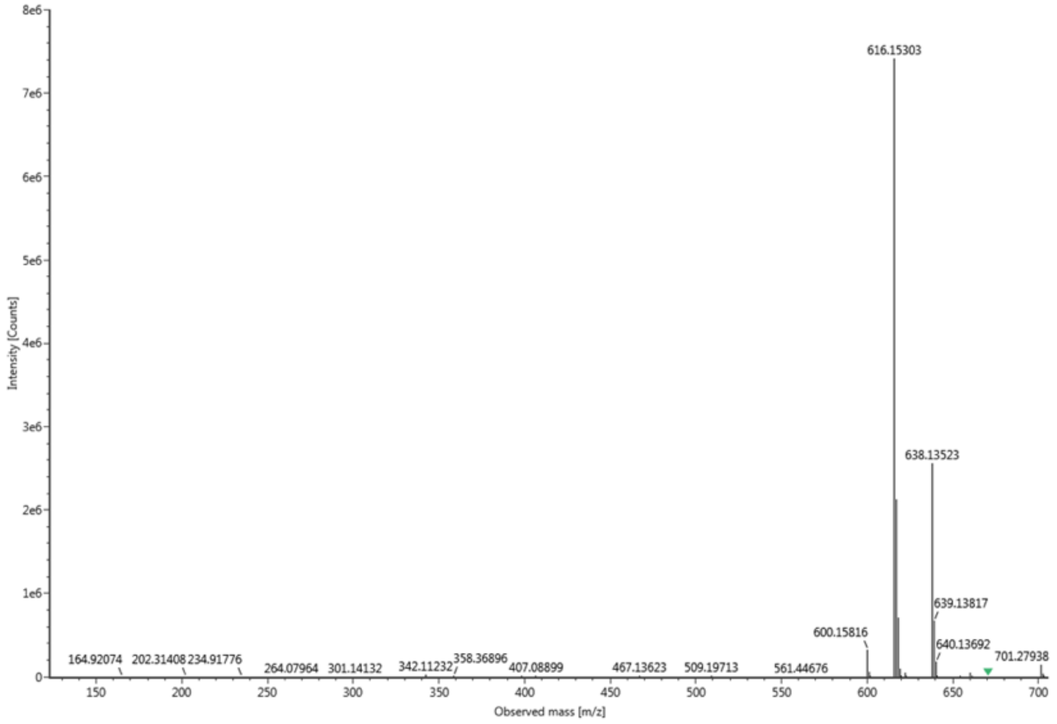

Compound NG: The 2.6 g of the compound, *N*, *N*-diisopropylethylamine 1.9 g, and HATU 3.2 g were added in 20 mL of *N*, *N*-dimethylaniline (DMA) and stirred at 25 ^{o}C for 0.5 h, and then 2.0 g of compound gemcitabine was added, stirred for another 10 h, and the reaction solution was concentrated under reduced pressure to yield a yellow solid. Afterward, the residual was extracted with ethyl acetate and water. And then, the combined organic layer was concentrated to give the desired product 0.9 g (yield, 20.7%), which was characterized by the NMR spectrum. ^{1}H NMR (400 MHz, DMSO-d6) δ (ppm): 10.09 (s, 1H), 8.21 – 8.19 (d, *J* = 3 Hz, 1H), 7.98 – 7.97 (t, *J* = 1.5 Hz, 1H), 7.96 – 7.55 (t, *J* = 1.5 Hz, 1H), 7.87 – 7.83 (dd, *J* = 3 Hz, 1H), 7.72 – 7.68 (t, *J* = 3 Hz, 2H), 7.26 – 7.24 (d, *J* = 3 Hz, 1H), 6.28 – 6.26 (d, *J* = 3 Hz, 1H), 6.15 – 6.11 (t, *J* = 3 Hz, 1H), 5.27 – 5.24 (t, *J* = 2 Hz, 1H), 4.35– 4.32 (t, *J* = 2 Hz, 2H), 4.16 – 4.13 (m, *J* = 1.5 Hz, 1H), 3.87 – 3.84 (m, *J* = 1.5 Hz, 1H), 3.78 – 3.75 (d, *J* = 4.5 Hz, 1H), 3.64 – 3.58 (m, *J* = 4 Hz, 1H), 2.39– 2.37 (t, *J* = 2 Hz, 2H), 1.71 – 1.68 (m, *J* = 1.5 Hz, 2H), 1.54 – 1.51 (m, *J* = 1.5 Hz, 2H), 1.30 – 1.28 (m, *J* = 1.5 Hz, 4H). ^{13}C NMR (101 MHz) δ (ppm):174.05, 162.88, 158.87, 154.20, 144.72, 137.24, 136.12, 130.02, 128.29, 125.51, 122.94, 120.36, 110.44, 95.92,

81.01, 71.41, 68.59, 68.37, 68.14, 58.78, 36.33, 27.91, 27.65, 24.76, 24.22. HRMS calcd for C24H27F2N5O10S, $[M+H]^+$ 616.15249; found 616.15303.

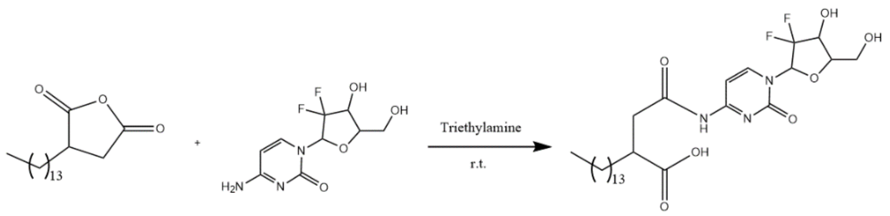

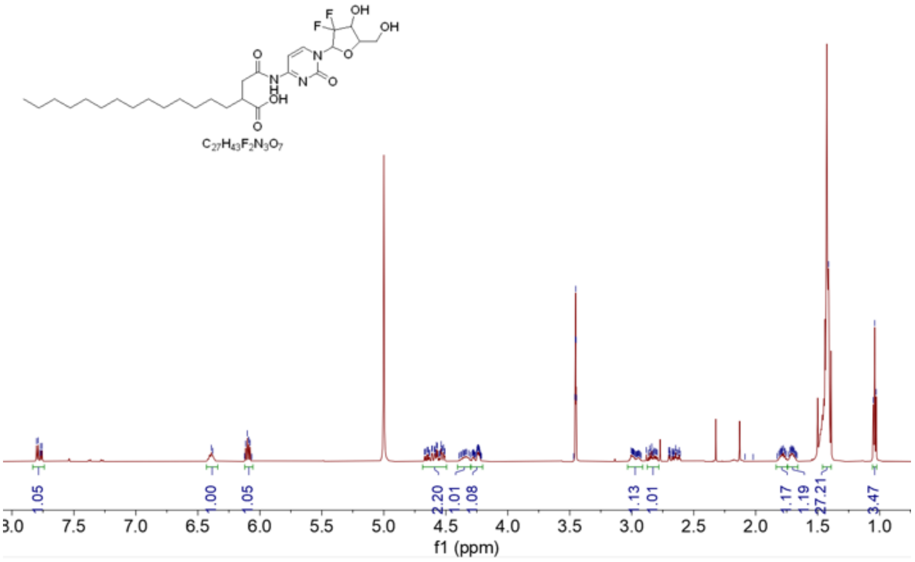

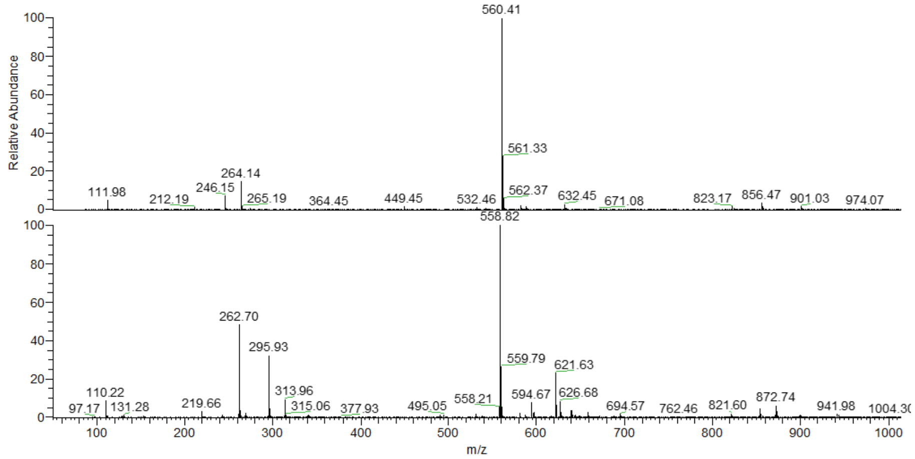

Figure S11 Synthesis procedure and characterization of C_{14}-Gem.

Gemcitabine 50 mg and tetradecyl succinic anhydride 85.6 mg were dissolved in 2 mL dry N, N-dimethylformamide (DMF) and added with triethylamine (1.5 eq). The mixture was stirred at room temperature for 24 h, extracted with ethyl acetate and water (1:1, v/v), and purified on

a silica gel column eluted by dichloromethane/methanol(Acetic Acid) [20:1(3‰), v/v] to obtain the desired product (yield, 28%), which was further characterized. ^{1}H NMR (600 MHz, Methanol-d4) δ 7.83 – 7.74 (m, 1H), 6.39 (d, J = 7.2 Hz, 1H), 6.12 – 6.06 (m, 1H), 4.69 – 4.49 (m, 2H), 4.34 (dq, J = 25.7, 9.7, 8.7 Hz, 1H), 4.30 – 4.20 (m, 1H), 3.03 – 2.91 (m, 1H), 2.88 – 2.78 (m, 1H), 1.83 – 1.74 (m, 1H), 1.74 – 1.66 (m, 1H), 1.41 (s, 27H), 1.04 (t, J = 7.0 Hz, 3H). $[M+H]^+$, 560.41, $[M-H]^-$, 558.82.

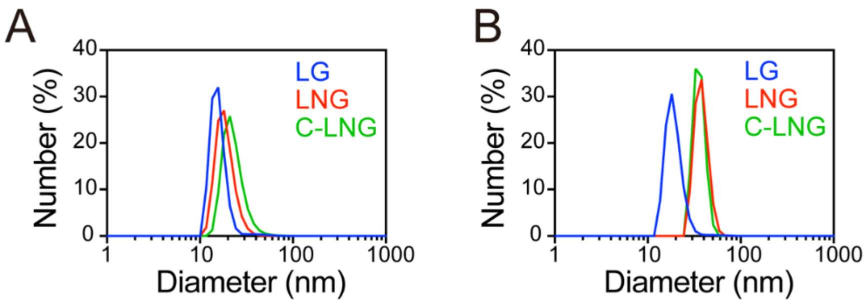

Figure S12 The particle size distribution of LG, LNG, and C-LNG upon their incubation in PBS (pH 7.4) (A) and PBS (pH 7.4)+10 mM GSH (B).

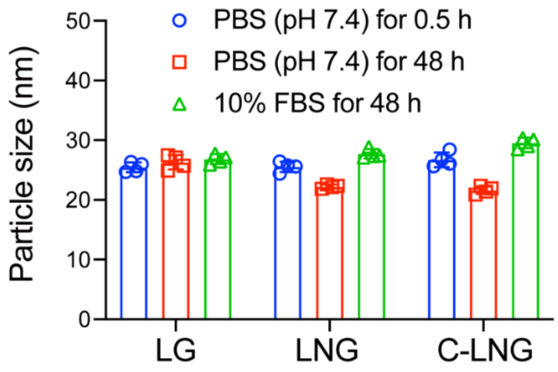

Figure S13 The mean diameter of LG, LNG, and C-LNG upon their incubation in PBS (pH 7.4) or PBS (pH 7.4) plus 10% FBS for 48 h. Data are means ± SD (n = 4).

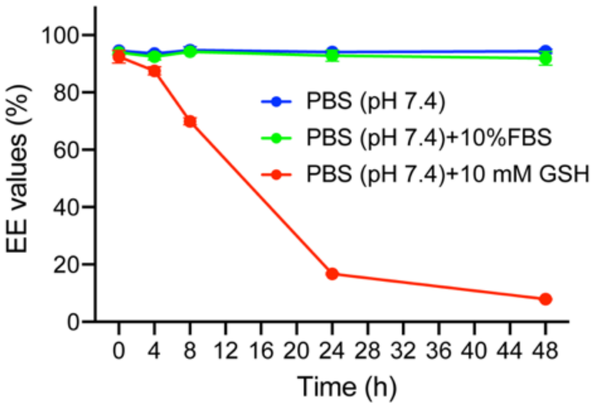

Figure S14 Stability and responsive drug release profile of C-LNG in different media by characterizing the EE values (n = 3).

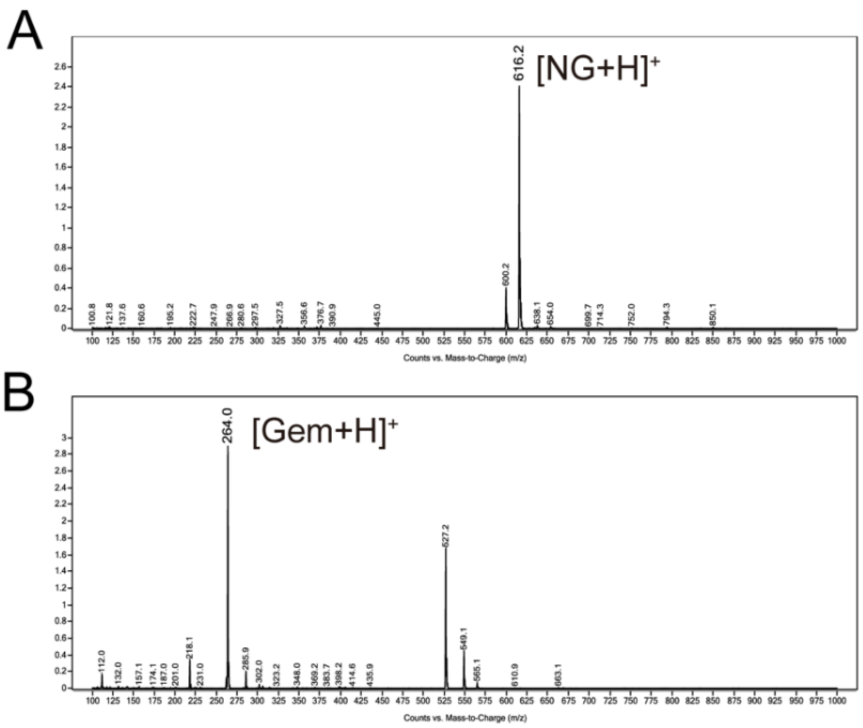

Figure S15 Degradation of NG in GSH-contain media. (A) ESI-MS analysis of NG, which was presented as $[NG + H]^+$. (B) ESI-MS analysis of Gem, which was presented as $[Gem + H]^+$.

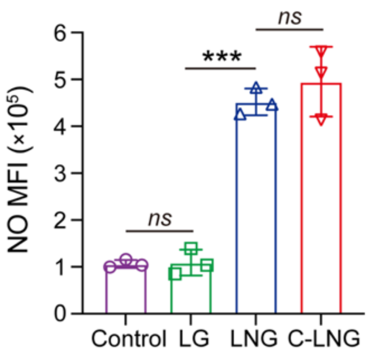

Figure S16 The NO production in GL261 cells treated with LG, LNG, and C-LNG (n = 3), $^{***}P < 0.001$.

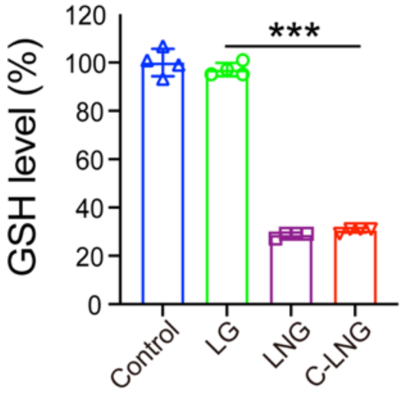

Figure S17 The GSH level in GL261 cells incubated with LG, LNG, and C-LNG (n = 4). Data are presented as mean ± SD. $^{***}P < 0.001$.

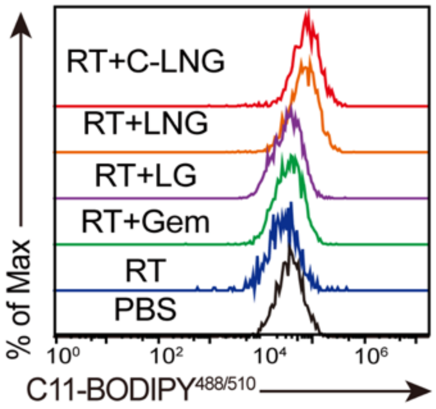

Figure S18 Flow cytometry analysis of lipid peroxidation in GL261 cells from each treatment.

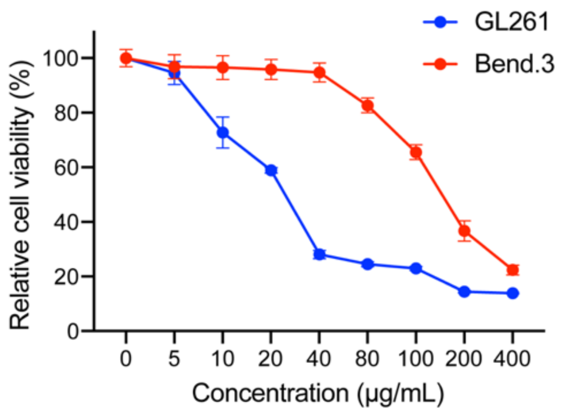

Figure S19 Cytotoxicity of C-LNG in GL261 cancer cells and Bend.3 normal cells at various drug concentrations (n = 4).

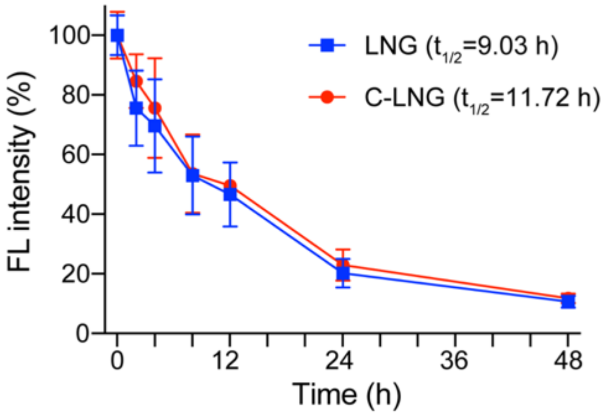

Figure S20 The in vivo pharmacokinetic profiles of DiD-labeled LNG and C-LNG in healthy mice (n=4).

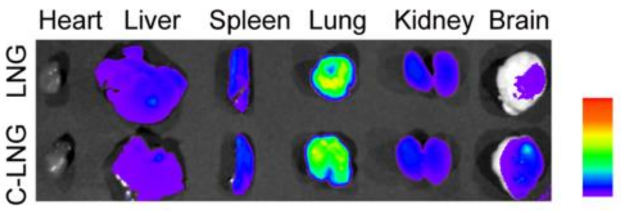

Figure S21 The ex vivo fluorescence images of the heart, liver, spleen, lungs, kidney, and brain at 12 h post-injection of LNG and C-LNG.

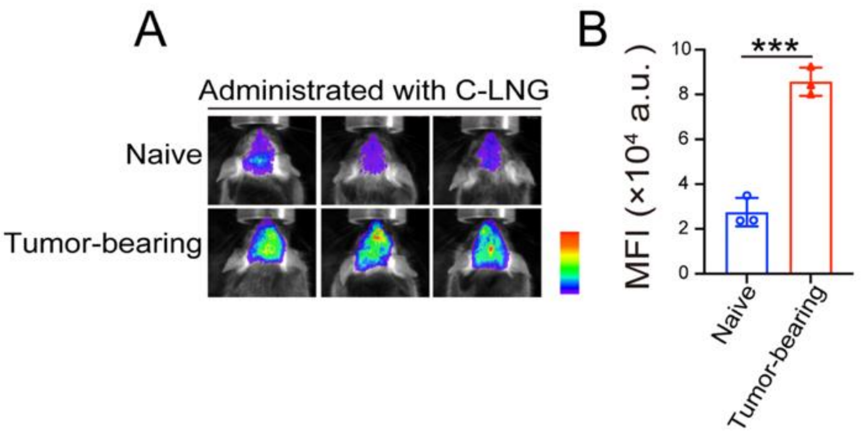

Figure S22 (A) *In vivo* fluorescence images and (B) quantitative analysis of C-LNG in healthy mice and GBM-bearing mice models at 12 h post-injection (n = 3). Data are presented as mean ± SD. $^{***}P < 0.001$.

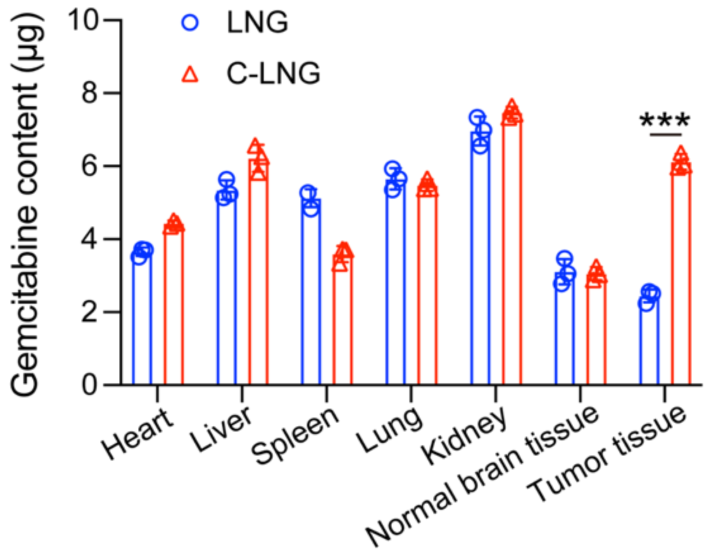

Figure S23 The quantified gemcitabine distribution in different major organs at 12 h after tail vein injection of LNG and C-LNG NPs (n = 3), $^{***}P < 0.001$.

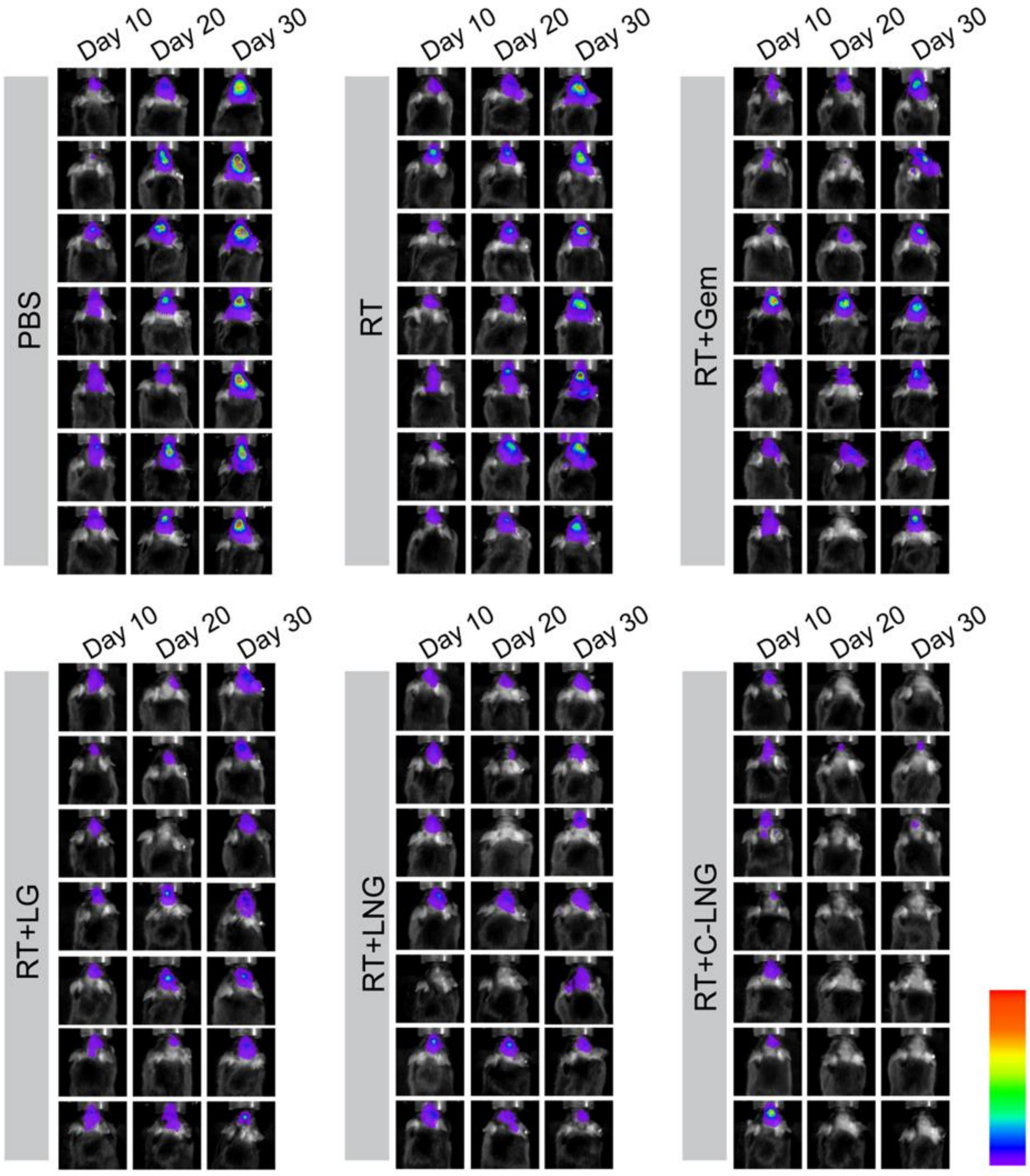

Figure S24 Bioluminescence images of GL261-luc induced orthotopic GBM tumor models from each treatment at the indicated time points (n = 7).

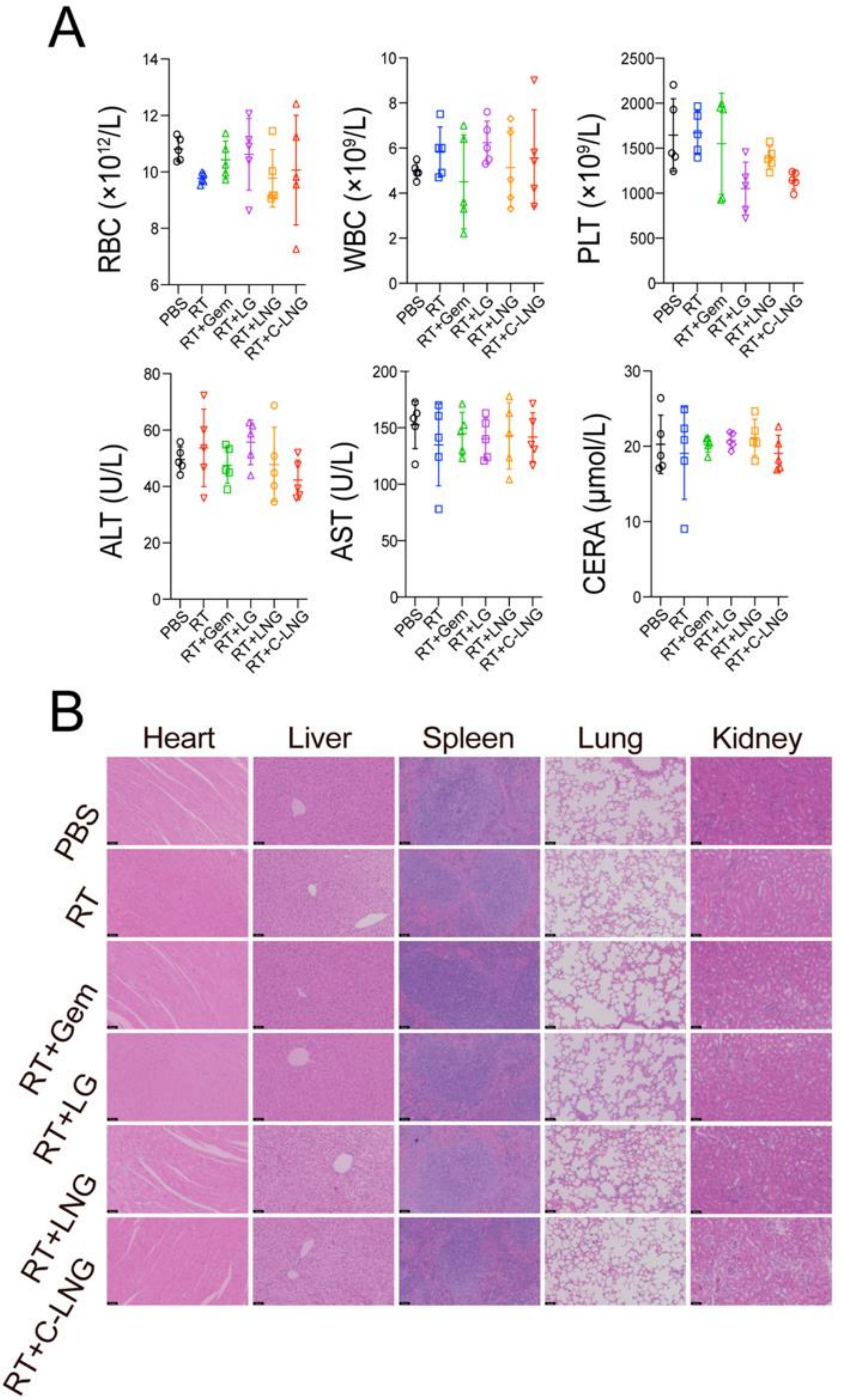

Figure S25 Biosafety assessments. (A) Hematology and blood biochemical tests were performed including red blood cells (RBC), white blood cells (WBC), platelet (PLT), aminotransferase (ALT), aspartate aminotransferase (AST), and creatinine (CREA) among all groups (n = 5). Data are presented as mean ± SD. (B) H&E staining of major organ tissues taken after various treatments. Scale bar: 50 μm.

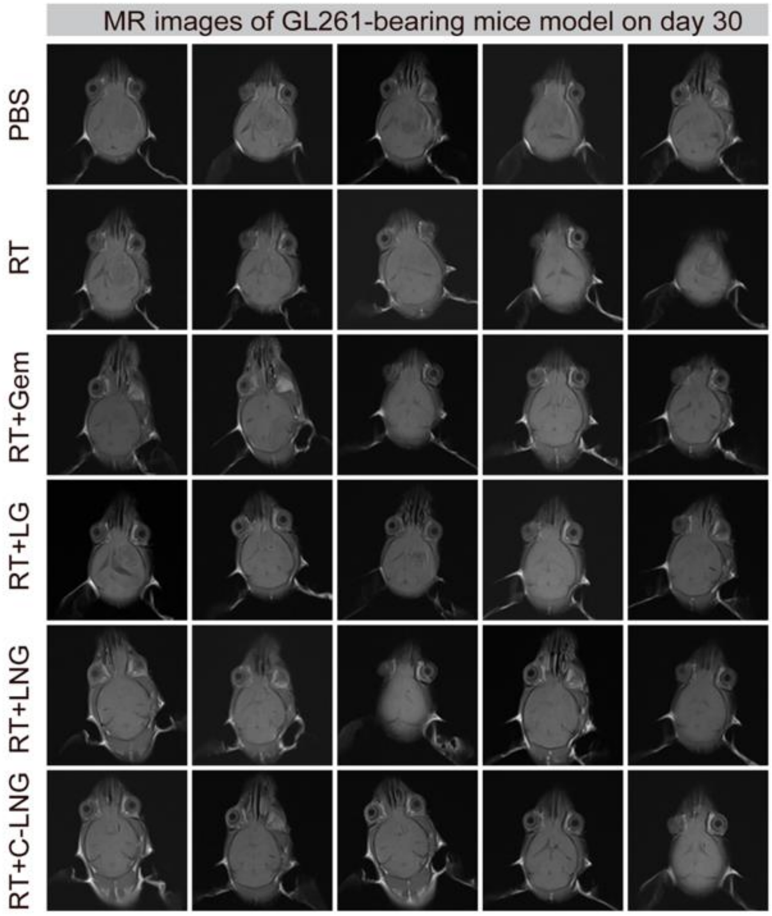

Figure S26 T1-weighted MR images of the GL261-luc induced GBM tumor models on day 30 post various treatments (n = 5).

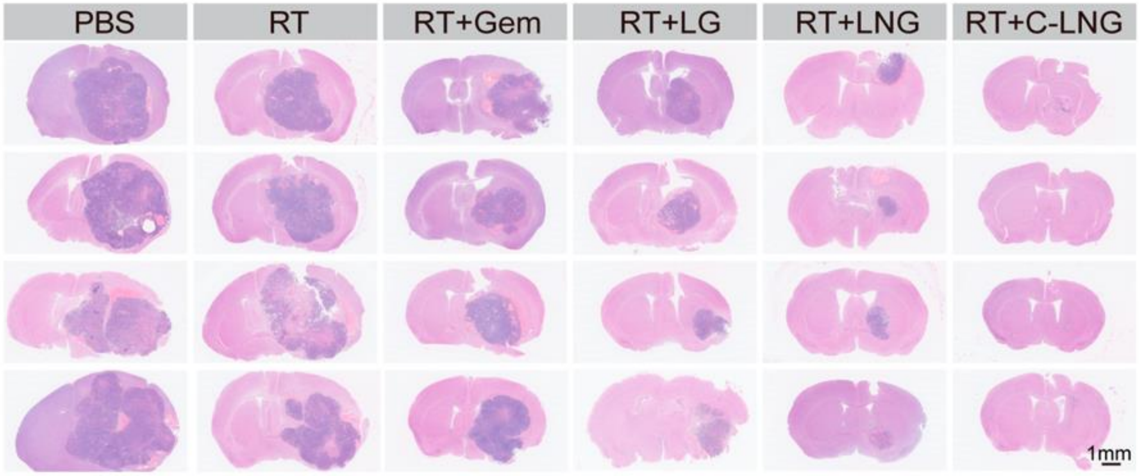

Figure S27 H&E staining images of the whole brain from GL261-luc induced GBM tumor models on day 30 post various treatments (n = 4).
